# Genome-wide imaging screen uncovers molecular determinants of arsenite-induced protein aggregation and toxicity

**DOI:** 10.1242/jcs.258338

**Published:** 2021-06-04

**Authors:** Stefanie Andersson, Antonia Romero, Joana Isabel Rodrigues, Sansan Hua, Xinxin Hao, Therese Jacobson, Vivien Karl, Nathalie Becker, Arghavan Ashouri, Sebastien Rauch, Thomas Nyström, Beidong Liu, Markus J. Tamás

**Affiliations:** 1 Department of Chemistry and Molecular Biology, University of Gothenburg, Box 462, SE-405 30 Göteborg, Sweden; 2 Institute of Biomedicine - Department of Microbiology and Immunology, Sahlgrenska Academy, University of Gothenburg, SE-405 30, Göteborg, Sweden; 3 Water Environment Technology, Department of Architecture and Civil Engineering, Chalmers University of Technology, SE-412 96 Göteborg, Sweden

**Keywords:** Arsenic, Protein misfolding, Protein aggregation, Transcription, Translation, Protein quality control, Proteostasis, Yeast

## Abstract

The toxic metalloid arsenic causes widespread misfolding and aggregation of cellular proteins. How these protein aggregates are formed *in vivo*, the mechanisms by which they affect cells and how cells prevent their accumulation is not fully understood. To find components involved in these processes, we performed a genome-wide imaging screen and identified *Saccharomyces cerevisiae* deletion mutants with either enhanced or reduced protein aggregation levels during arsenite exposure. We show that many of the identified factors are crucial to safeguard protein homeostasis (proteostasis) and to protect cells against arsenite toxicity. The hits were enriched for various functions including protein biosynthesis and transcription, and dedicated follow-up experiments highlight the importance of accurate transcriptional and translational control for mitigating protein aggregation and toxicity during arsenite stress. Some of the hits are associated with pathological conditions, suggesting that arsenite-induced protein aggregation may affect disease processes. The broad network of cellular systems that impinge on proteostasis during arsenic stress identified in this current study provides a valuable resource and a framework for further elucidation of the mechanistic details of metalloid toxicity and pathogenesis.

This article has an associated First Person interview with the first authors of the paper.

## Abstract (precis)

**Summary:** Identification of cellular systems that impinge on protein homeostasis during arsenite stress in yeast, showing that transcriptional and translational control is central for mitigating protein aggregation and toxicity.

## INTRODUCTION

The processes that control protein synthesis, folding, localization, abundance and degradation are crucial for the proper functioning of cells and organisms. The ability of the cell to maintain a functional proteome (protein homeostasis or proteostasis) declines during ageing, which may lead to the accumulation of damaged, misfolded and aggregated proteins. The proteome is also threatened by environmental stress conditions that promote rapid and extensive protein misfolding and aggregation. Excessive protein misfolding and aggregation can cause cellular or organismal damage, as exemplified by the many pathological conditions that are associated with defective proteostasis, including neurodegenerative and age-related disorders such as Alzheimer's disease (AD) and Parkinson's disease (PD) (Goloubinoff, 2016; Hipp et al., 2019; Sala et al., 2017). How different aggregated protein species cause or contribute to toxicity or pathology is poorly understood.

Cells use a battery of protein quality control (PQC) mechanisms to ensure proteostasis. Molecular chaperones assist the folding of proteins into their functional conformation or help misfolded proteins to regain their native structures. Molecular chaperones are also integral parts of protein degradation systems, such as the proteasome, the autophagy pathway and the lysosome/vacuole, that clear cells from aberrant protein conformers (Goloubinoff, 2016; Hipp et al., 2019; Sala et al., 2017). Misfolded and aggregated proteins may be directed to specific subcellular deposition sites, as a means to reduce their toxicity (Miller et al., 2015). Cells may also use the controlled formation of protein aggregates for various physiological purposes, such as storage of peptide and protein hormones (Maji et al., 2009), regulation of cell cycle restart after stress (Saad et al., 2017), and microbial adhesion, biofilm formation and host invasion (Fowler et al., 2007; Gebbink et al., 2005). The potential toxicity or benefit of protein aggregates highlights the importance of proteostasis during physiological conditions as well as during ageing, pathological conditions and stress.

Human exposure to poisonous metals is increasing in many parts of the world, and chronic exposure is associated with certain protein folding-associated diseases including AD and PD (Caudle et al., 2012; Chin-Chan et al., 2015; Gong and O'Bryant, 2010; Wang and Du, 2013). While the toxicity of many metals is undisputed, their molecular modes of action have remained unclear. Recent *in vitro* and *in vivo* studies revealed that toxic metals profoundly affect proteostasis (Holland et al., 2007; Jacobson et al., 2012, 2017; Ramadan et al., 2009; Sharma et al., 2008; Tamás et al., 2018; Tamás et al., 2014). Arsenic, in the form of trivalent arsenite [As(III)] (Ibstedt et al., 2014; Jacobson et al., 2012), and cadmium (Jacobson et al., 2017) have been shown to cause widespread protein aggregation in living yeast cells primarily by targeting nascent proteins. *In vitro* and *in vivo* data suggest that As(III)- and cadmium-aggregated protein species may form seeds that increase the misfolding and aggregation of other susceptible proteins. These studies also suggested that misfolding and aggregation of nascent proteins represents an important component of mode of toxic action for arsenite and cadmium (Jacobson et al., 2012, 2017). Toxicity of chromium, in the form of Cr(VI), is partly a result of enhanced mRNA mistranslation leading to the accumulation of misfolded and aggregated proteins (Holland et al., 2007). Despite these examples, our current knowledge of the molecular basis of metal stress-induced protein aggregation in living cells and how cells regulate PQC systems to avoid toxicity is incomplete. In this study, we used high-content microscopy to identify a broad network of cellular systems that impinge on proteostasis and cell viability during arsenite stress. Follow-up experiments highlight the importance of accurate transcriptional and translational control for mitigating arsenite-induced protein aggregation and toxicity.

## RESULTS

### Genome-wide imaging screen uncovers factors that impinge on proteostasis during As(III) stess

**Fig. 1.**
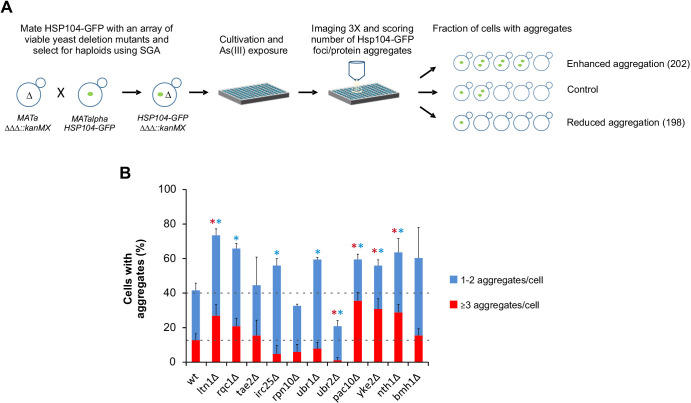
**High-content imaging screen identifies molecular determinants of As(III)-induced protein aggregation.** (A) Schematic representation of the workflow. Hits were divided into two categories: mutants that accumulated more aggregates than the wild type (enhanced aggregation) and mutants with fewer aggregates than the wild type (reduced aggregation). (B) Hsp104–GFP distribution was scored after 3 h of As(III) exposure (0.5 mM) in selected mutants. The fractions of cells containing 1-2 aggregates/cell and ≥3 aggregates/cell were determined by visual inspection of ∼200 cells per strain. Error bars represent standard deviations (s.d.) from two (mutants) and six (wild type, wt) independent biological replicates. Error bars on the top concern the total fraction of cells with aggregates; those on the red bars concern the fraction of cells with ≥3 aggregates/cell. *P<0.01 compared with wild type (unpaired, two-tailed Student's *t*-test; blue, total fraction of cells with aggregates; red, ≥3 aggregates/cell).

To identify factors that impinge on proteostasis during As(III) exposure, we performed a high-content imaging screen in the budding yeast *Saccharomyces cerevisiae*. A GFP-tagged version of Hsp104, a molecular chaperone that binds to and disassembles protein aggregates (Glover and Lindquist, 1998), was incorporated into a genome-wide collection of viable deletion mutants using a synthetic genetic array (SGA) approach (Fig. 1A). Hsp104–GFP can be used as a visual marker during As(III) exposure as it redistributes from a diffuse cytosolic localization to specific foci that represent protein aggregates (Jacobson et al., 2012). We cultivated and exposed this strain collection to As(III) using high-throughput robotic handling in a microtiter plate format. The percentage of cells containing Hsp104–GFP foci/protein aggregates was scored using automated image analysis. To allow identification of mutants that accumulate either more or fewer protein aggregates than the wild-type control, we chose an As(III) concentration (0.25 mM) and a time point (2 h) at which ∼40% of the wild-type cells contained Hsp104–GFP foci. We counted cells that had one or two aggregates per cell and those with three or more aggregates per cell, and the obtained values for each mutant were compared to those for wild-type cells. Mutants that deviated significantly (*P*<0.05) in aggregate levels (fraction of cells with aggregates) from the wild type were selected for further analysis (Tables S1, S2). In this way, we found 202 mutants that accumulated more aggregates (i.e. showing enhanced aggregation) and 198 mutants with fewer aggregates (reduced aggregation) than the wild type (Fig. 1A).

The large number of hits suggests that yeast devotes a substantial fraction (the ∼400 hits represent ∼8% of non-essential *S. cerevisiae* genes) of its genome into PQC during As(III) stress. Among the hits, several were previously known or expected to affect proteostasis (see below), validating the screening approach. To directly test the accuracy of the screen, we manually re-created 11 strains by crossing individual mutants selected from our hit list with an Hsp104–GFP strain, exposed the resulting strains to 0.5 mM As(III) [a higher As(III) concentration is needed to obtain similar aggregation levels in flasks versus microtiter plates], and scored their respective protein aggregation levels. Eight out of the 11 strains deviated significantly (*P*<0.01) from the protein aggregation levels in wild-type cells (Fig. 1B). Using other assays, we confirmed another 8 mutants out of 12 tested (see Figs 6 and 7), suggesting an overall accuracy of at least 70% (true positives). The gene products that are directly involved in proteostasis remain to be distinguished from those with an indirect effect.

### Correlation to previously reported genome-wide screens

To assess whether the identified mutants are affected in PQC, we compared our hits to sets of mutants that display altered activity of a reporter containing binding sites (so-called heat shock elements or HSEs) for yeast heat shock factor 1 (Hsf1) at 25°C (Brandman et al., 2012). Hsf1 controls expression of genes dedicated to protein folding under both basal and heat-shock conditions (Solís et al., 2016). We observed significant overlaps between the reduced aggregation set and a set of 92 mutants with low HSE activity (15 genes, *P*<10^−6^) as well as between the enhanced aggregation set and 34 mutants with high HSE activity (seven genes, *P*=0.0005) (Fig. S1A, Table S3). In contrast, the overlaps were insignificant between the enhanced aggregation and low HSE activity sets (three genes, *P*=0.19), and the reduced aggregation and high HSE activity sets (three genes, *P*=0.12). Thus, the identified mutants do not appear to have a general PQC defect.

Stress granules (SGs) and processing bodies (PBs) are cytosolic structures that contain mRNA and RNA-binding proteins that form during severe stress conditions (Buchan and Parker, 2009) and there is evidence of cross-talk between SGs and PBs and the PQC machinery (Alberti and Hyman, 2021; Cherkasov et al., 2013). Comparing our datasets with sets of mutants having either elevated (92 genes) or diminished (102 genes) SG formation during glucose deprivation (Yang et al., 2014) revealed significant overlaps in all tested conditions (Fig. S1B, Table S3). There was also significant overlap between the enhanced aggregation gene set and a set of mutants (102 genes) showing constitutive SG formation (eight genes, *P*=0.04) (Buchan et al., 2013) (Fig. S1C, Table S3). In contrast, the overlap was poor between our gene sets and sets of mutants having either elevated or diminished PB levels under non-stress conditions (Buchan et al., 2013) (Fig. S1D, Table S3). Some SG and PB components were present in our datasets (e.g. Pbp1, Dhh1 and Xrn1) but most core SG and PB proteins were absent. The majority of Hsp104–GFP-containing foci do not colocalize with SGs and PBs during As(III) exposure in *S. cerevisiae* (Jacobson et al., 2012), suggesting that formation of Hsp104–GFP foci and SGs and PBs might be largely distinct. Nevertheless, some of the genes involved in PQC during As(III) stress may also modulate SG assembly and/or disassembly.

We next compared our datasets to a set of 104 mutants with defects in assembly of large inclusions during heat stress (Babazadeh et al., 2019) and found a substantial overlap with the enhanced aggregation gene set (27 genes, *P*<10^−14^) (Fig. S1E, Table S3). In contrast, the overlap is insignificant between the reduced aggregation and defective inclusion assembly sets (2 genes, *P*=0.10). This raises the possibility that yeast cells use partially overlapping machineries to regulate aggregate assembly and/or clearance during As(III) and heat stress. Taken together, these analyses suggest that our datasets contain factors that may act specifically during As(III)-induced protein aggregation as well as general factors that act under proteotoxic stress.

### Loss of functions related to transcription and translation lead to reduced protein aggregation levels

**Fig. 2.**
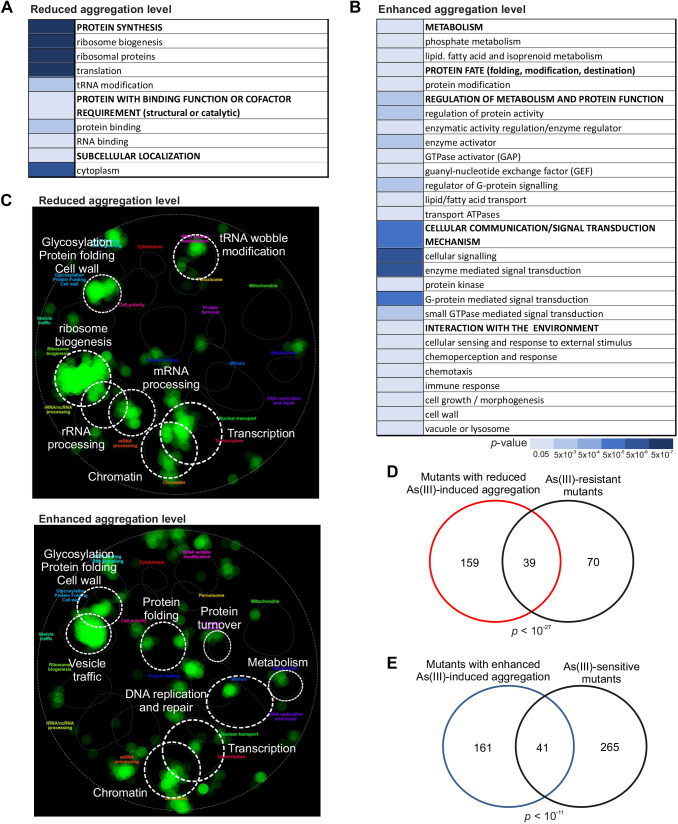
**Characterization of the hits.** Functional categories of hits that were significantly enriched (FDR<0.05) among mutants with reduced (A) and enhanced (B) aggregation. (C) The identified hits belong to distinct functions. Each hit was mapped onto the global yeast genetic interaction network and visualized using CellMap. Correlation between protein aggregation and As(III) sensitivity or resistance. (D,E) The Venn diagrams show the overlap between (D) mutants with reduced aggregation and As(III)-resistant mutants and between (E) mutants with enhanced aggregation and As(III)-sensitive mutants. Significance of the overlaps was calculated by the hyper-geometric test and the corresponding *P*-values are indicated.

We next investigated whether specific categories of protein functions were over represented in our datasets. The set of mutants with reduced aggregation levels was primarily enriched for genes with functions in protein biosynthesis, protein and RNA binding, and for cytosolic proteins (Fig. 2A; Table S1). A large proportion of the hits are part of highly connected networks [protein–protein interaction (PPI) enrichment *P*-value <10^−16^] involved in cytoplasmic translation (e.g. ribosomal proteins), rRNA modification and processing, and ribosome assembly, as well as several members of the elongator (ELP) complex, which is primarily involved in tRNA modifications (Johansson et al., 2018) (Fig. S2; Table S1). A set of hits with reduced aggregation levels were related to As(III) import, including Fps1, the aquaglyceroporin that mediates As(III) entry into cells (Wysocki et al., 2001), and positive regulators of Fps1 [Rgc1, Rgc2 (also known as Ask10) (Beese et al., 2009) and Slt2 (Ahmadpour et al., 2016)] (Fig. S2). Mapping each hit onto the global yeast genetic interaction network (Costanzo et al., 2016; Usaj et al., 2017) showed that the hits are clustered in distinct functions related to chromatin, transcription, ribosome biogenesis, rRNA processing, mRNA processing and tRNA wobble modification, as well as glycosylation, protein folding and the cell wall (Fig. 2C). Since As(III) targets nascent proteins for aggregation (Jacobson et al., 2012), we asked whether mutants with fewer aggregates have decreased translational activity. Slow growth is accompanied by a reduction of translation-related proteins (Metzl-Raz et al., 2017) and probably by lower protein synthesis rates. We found a significant overlap between a set of 122 mutants that grow slowly in minimal synthetic complete medium (Zackrisson et al., 2016) and the reduced aggregation set (19 genes, *P*<10^−7^) (Fig. S1F, Table S3). The overlap between slow-growing and enhanced aggregation sets was less prominent (nine genes, *P*=0.05). Thus, ongoing translation may result in protein aggregation during As(III) exposure. Together, the hits support earlier observations that processes related to translation impinge on proteostasis during As(III) stress (Guerra-Moreno et al., 2015; Ibstedt et al., 2014; Jacobson et al., 2012). Our analysis also identified protein modules that were previously not linked to PQC, including proteins with functions in mRNA modification, transcription and chromatin organization (Fig. S2). Thus, transcription-related processes may contribute to proteostasis during As(III) stress (see below).

### Mutants with enhanced levels of protein aggregation are enriched in a range of cellular functions

The set of mutants with enhanced aggregation levels was enriched in a range of biological functions including metabolism (primarily phosphate and lipid metabolism), protein fate (folding, modification, destination), regulation of metabolism and protein function, cellular communication and signaling, as well as interaction with the environment (Fig. 2B; Table S2). The hits are part of highly connected networks (PPI enrichment *P*-value 10^−14^) associated with protein folding and degradation, signaling, transcription, and lipid/fatty acid metabolism (Fig. S3). A set of the hits were related to As(III) export or intracellular sequestration including the As(III) exporter Acr3 (also known as Arr3) (Wysocki et al., 1997), Yap8 (also known as Arr1), a transcription factor that regulates *ACR3* expression (Kumar et al., 2016; Wysocki et al., 2004), Ycf1, a transporter that catalyzes As(III) sequestration into vacuoles (Ghosh et al., 1999), Yap1, the transcription factor that regulates *YCF1* expression (Wysocki et al., 2004), and Ybp1, a positive regulator of Yap1 (Veal et al., 2003). Mapping the hits onto the global yeast genetic interaction network identified clusters of functions related to chromatin, transcription, metabolism, DNA replication and repair, protein folding and turnover and vesicle trafficking, as well as glycosylation, protein folding and the cell wall (Fig. 2C). Protein folding-associated modules included members of the prefoldin complex, which is involved in actin and tubulin folding, as well as in transcription elongation (Payán-Bravo et al., 2018), and members of the GET complex, which promotes insertion of tail-anchored proteins into the ER membrane (Fig. S3). One of the GET complex proteins, Get3, also functions as a holdase chaperone under oxidative stress conditions (Powis et al., 2013; Voth et al., 2014). The presence of protein folding-related genes in the enhanced aggregation set supports previous findings that As(III) interferes with protein folding processes *in vivo* (Ibstedt et al., 2014; Jacobson et al., 2012). Protein turnover-associated hits included members of the ribosomal quality control (RQC) complex, which mediates the ubiquitylation of ribosome-associated aberrant nascent polypeptides (Brandman et al., 2012; Defenouillère and Fromont-Racine, 2017), components of the ubiquitin-proteasome pathway and proteins involved in intracellular trafficking, including autophagy-related proteins. Thus, clearance of As(III)-induced protein aggregates may involve both the ubiquitin–proteasome and the autophagy pathways, in line with previous reports (Guerra-Moreno et al., 2015; Jacobson et al., 2012). A large number of genes previously not linked to PQC were also identified. For example, network analyses pinpointed protein modules involved in signaling [Snf1 and glucose/cAMP signaling pathways, the Hog1 mitogen-activated protein kinase (MAPK) pathway and G-protein-mediated signaling], and transcription, including the Rpd3 histone deacetylase complex (Fig. S3). Hence, signal transduction and transcriptional regulatory pathways may impinge on PQC during stress. A few genes with translation-related functions were found in the enhanced aggregation set, whereas the majority of protein synthesis-related genes were present in the reduced aggregation set (Fig. S2). It is possible that this subset of proteins has specific functions during translation and that their absence might result in imperfect or increased translation, which in turn may lead to increased protein misfolding. Accordingly, cells lacking Asc1, a negative regulator of translation (Gerbasi et al., 2004), exhibited enhanced aggregation during As(III) stress (Table S2; Fig. S3).

### Correlation between protein aggregation levels and As(III) sensitivity

Protein aggregates may be toxic or beneficial. To address, in an unbiased manner, whether the aggregates formed during As(III) stress contribute to toxicity, we compared our gene sets to mutants identified in genome-wide As(III) sensitivity and resistance screens. We found a significant overlap (39 genes, *P*<10^−27^) between mutants with reduced aggregation and a set of 109 As(III)-resistant mutants (Pan et al., 2010) (Fig. 2D; Table S3). Likewise, we observed a significant overlap (41 genes, *P*<10^−11^) between the mutants with enhanced aggregation and 306 As(III)-sensitive mutants (Thorsen et al., 2009) (Fig. 2E; Table S3). In contrast, the overlaps were small between the enhanced aggregation and As(III) resistance sets (seven genes, *P*=0.10), and between the reduced aggregation and As(III) sensitivity sets (five genes, *P*=0.005). Thus, on a genome-wide scale, enhanced protein aggregation correlated with As(III) sensitivity, whereas As(III) resistance correlated with reduced aggregation levels. These findings support the notion that protein misfolding and aggregation contribute to the toxicity of this metalloid. We note that the majority of the genes in the respective screens are not overlapping, indicating that several toxicity mechanisms act in parallel.

### As(III) does not induce transcription errors

**Fig. 3.**
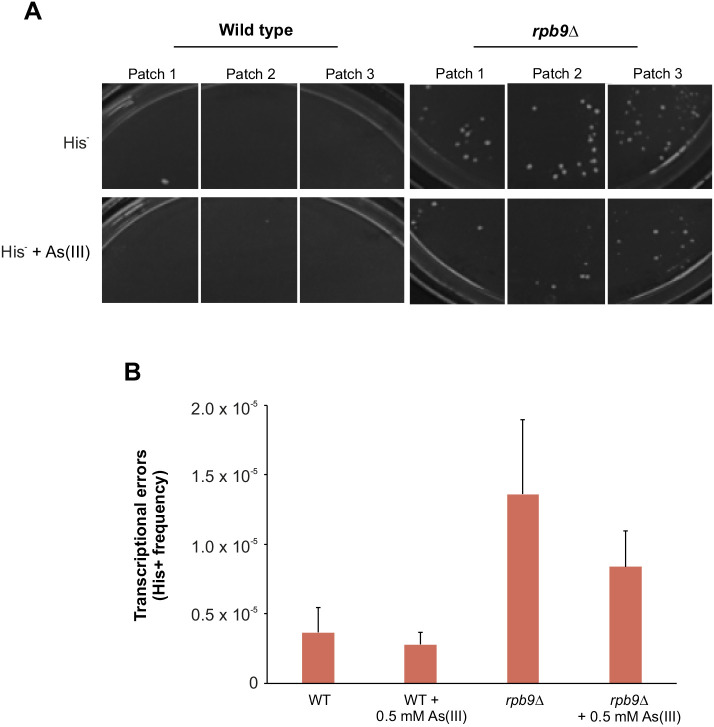
**As(III) does not induce transcription errors.** (A) P*_GAL1_*-*cre*-*Y324C* strains (wild-type and *rpb9*Δ) were grown on YPD agar plates overnight and replica-plated onto synthetic medium lacking histidine (His^−^) with or without 0.5 mM As(III). Transcription error results in growth of colonies within a patch on His^−^ plates. Three representative patches are shown for each strain. Pictures were taken after 3 days of growth at 30°C. (B) Mean±s.e.m. (*n*=3) frequency of His^+^ cells relative to total viable cells. P*_GAL1_*-*cre*-*Y324C* cells were grown overnight in liquid YPD medium and plated on His^−^ and YPD plates in 10-fold dilutions. Individual colonies were scored after 3 days of growth at 30°C.

Several transcription-related genes were present in both the reduced and enhanced aggregation sets (Fig. 2). Most of these genes have no previous association with PQC and the impact of transcriptional control on proteostasis is largely unexplored. One way As(III) could trigger protein aggregation is by causing errors during transcription. Transcription errors have been shown to cause proteotoxic stress and to shorten the lifespan of yeast (Vermulst et al., 2015). To test whether As(III) affects the frequency of transcriptional errors, we used an established genetic assay that detects transcription errors by a Cre-dependent rearrangement of a *HIS3*-based reporter gene (Irvin et al., 2014). Patches of cells were grown on YPD medium and then replica plated onto medium lacking histidine (His^−^). On His^−^ plates, colonies of His^+^ cells that arise due to transcription errors can grow within the patch. Under non-stress conditions, few wild-type cells grew on His^−^ medium (Fig. 3A) indicating a low transcription error rate. In the presence of As(III), only a few colonies grew on His^−^ plates indicating that As(III) does not promote transcription errors. We repeated the experiment with cells lacking Rpb9 given that this mutant displays reduced transcription fidelity and elevated error frequency (Irvin et al., 2014). Accordingly, many colonies of *rpb9*Δ grew on His^−^ plates under non-stress conditions (Fig. 3A). As for the wild type, As(III) did not affect the number of *rpb9*Δ colonies on His^−^ medium compared to the number seen in non-stress conditions. In addition to these qualitative assays, we performed quantitative assays where we quantified the mean frequency of His^+^ cells relative to the total number of viable cells. Again, there was no significant impact of As(III), neither in wild-type nor *rpb9*Δ cells (Fig. 3B). Thus, As(III) does not induce transcription errors.

### Global transcription affects protein aggregation levels during As(III) stress

**Fig. 4.**
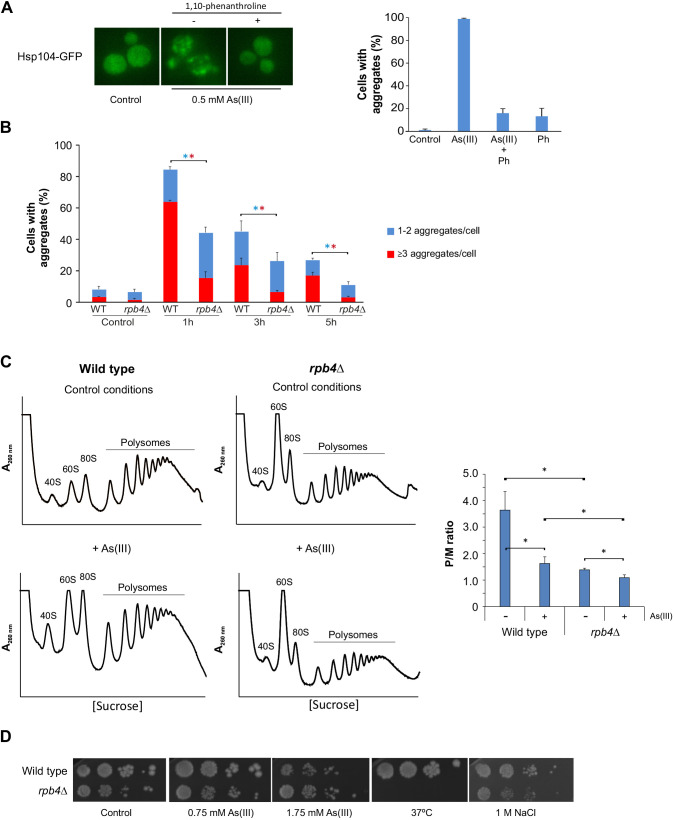
**Global transcription affects protein aggregation levels during As(III) stress.** (A) Hsp104–GFP localization was monitored in wild-type cells before and after 1 h exposure to 0.5 mM As(III) in the absence or presence of 0.1 mg/ml 1,10-phenantroline (Ph). The fractions of cells containing aggregates were determined by visual inspection of 20–135 cells per condition. Results are mean±s.d. from three independent biological replicates (*n*=3). Left panel, representative images are shown. Right panel, quantification of protein aggregation. (B) Sis1–GFP distribution was scored in wild-type (WT) and *rpb4*Δ cells by fluorescence microscopy before and after exposure to 0.5 mM As(III). The fractions of cells containing 1–2 aggregates/cell and ≥3 aggregates/cell were determined by visual inspection of 246–370 cells per condition and time-point Results are mean±s.d. from three independent biological replicates (*n*=3). Error bars on the top concern the total fraction of cells with aggregates, whilst those on the red bars concern the fraction of cells with ≥3 aggregates/cell. *P<0.05 (unpaired, two-tailed Student's *t*-test; blue, total fraction of cells with aggregates; red, ≥3 aggregates/cell). (C) Polysome profiles were obtained from cells cultivated in SC medium for at least 3 h to reach exponential phase, and then cells were further maintained in or treated with 0.75 mM As(III) for 1 h. The *A*_260nm_ profiles after gradient fractionation are shown and the ribosomal subunits (40S and 60S), monosomes (80S) and polysomes are indicated. Three biologically independent replicates were performed (*n*=3) and a representative profile is shown. Right panel, the mean±s.d. P/M ratio is shown. *P<0.05 (unpaired, two-tailed Student's *t*-test). (D) 10-fold serial dilutions of the indicated cells were plated onto YPD agar plates with or without As(III) or NaCl. Growth was monitored after 2–3 days at 30°C or 37°C. Images representative of three experiments.

A subset of hits with reduced aggregation encode proteins that act as positive regulators of transcription (Fig. S2), raising the possibility that a decrease in global transcription, and hence protein synthesis, limits protein aggregation levels during As(III) stress. To test this, we incubated cells with As(III) and the transcription inhibitor 1,10-phenanthroline, added either separately or together, and monitored Hsp104–GFP distribution. Indeed, 1,10-phenanthroline strongly attenuated As(III)-induced protein aggregation, and few Hsp104–GFP foci were detected in the presence of this chemical (Fig. 4A). Next, we scored protein aggregation levels in cells lacking Rpb4, a subunit of the RNA polymerase II enzyme; *rpb4*Δ cells have decreased global transcription while they maintain a balanced level of mRNA because it is globally stabilized (Garrido-Godino et al., 2016). The *rpb4*Δ mutant was transformed with Sis1–GFP, an essential Hsp70 co-chaperone (Yan and Craig, 1999) that associates with aggregation-prone proteins in proteotoxic stress conditions (Malinovska et al., 2012; Park et al., 2013). As seen with Hsp104–GFP, 1 h of As(III) exposure resulted in Sis1–GFP redistribution to distinct cytosolic foci, with the majority of cells containing several foci (Fig. 4B). The total fraction of cells with foci decreased over time, as did the proportion of cells containing three or more Sis1–GFP foci/cell. Interestingly, *rpb4*Δ had substantially fewer aggregates than the wild type during exposure (Fig. 4B). Thus, a global reduction of transcription by chemical or genetic means can mitigate protein aggregation during As(III) stress. To test whether a global reduction of transcription is accompanied by diminished translation, we performed polysome-profiling assays. For wild-type cells, a general repression of translation initiation occurs in response to As(III), as evidenced by increased levels of ribosomal subunits (40S and 60S) and monosomes (80S), and by decreased levels of polysomes (Fig. 4C). Quantification of the polysome-to-monosome ratio (P/M) in wild-type cells showed a ∼2-fold reduction in translation during exposure. Notably, *rpb4*Δ had lower translational activity than the wild type both in the absence and presence of As(III) (Fig. 4C). The P/M ratio for *rpb4*Δ was ∼2-fold lower than for wild-type cells in the absence of stress and was further reduced by ∼30% during As(III) exposure. Importantly, *rpb4*Δ grew better in As(III)-containing medium than wild-type cells (Fig. 4D) indicating that reduced protein synthesis is beneficial during As(III) exposure. In contrast, *rpb4*Δ was sensitive to increased temperature (37°C), a condition that causes misfolding of nascent as well as native proteins. The *rpb4*Δ mutant was also sensitive to high osmolarity (1 M NaCl), a condition that does not affect protein folding. Taken together, for *rpb4*Δ cells, a reduction in global transcription is accompanied by low translational activity that likely protects the *rpb4*Δ proteome from As(III)-induced misfolding and aggregation.

### Loss of transcriptional control impacts protein aggregation levels during As(III) stress through distinct mechanisms

It is unlikely that all transcription-related hits affect global transcription, as shown for Rpb4. Instead, the majority of these proteins are expected to have limited impact on global mRNA levels and likely affect proteostasis through distinct mechanisms.

#### Positive regulators of transcription

**Fig. 5.**
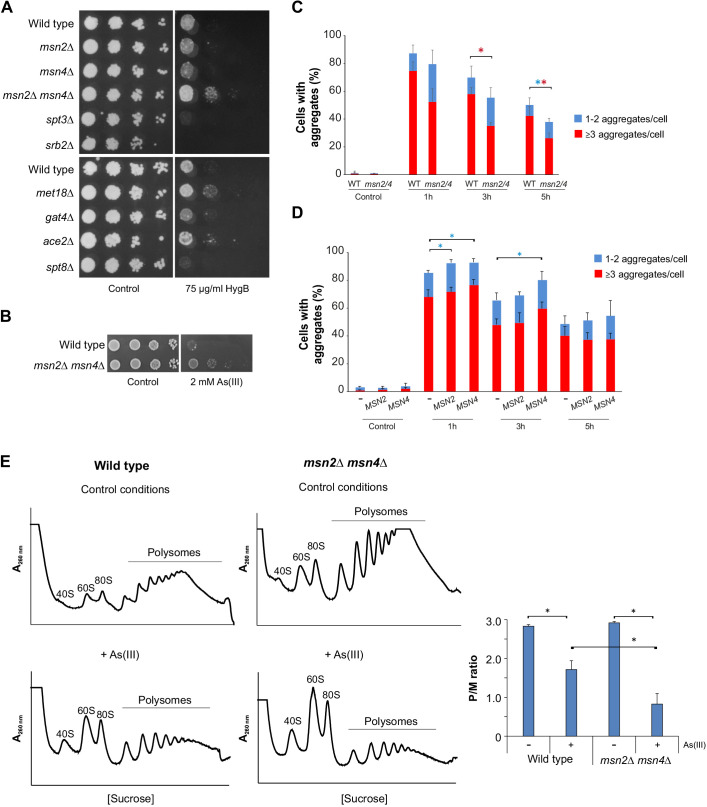
**Loss of positive regulators of transcription leads to reduced protein aggregation.** (A) 10-fold serial dilutions of the indicated strains were plated onto YPD agar plates with or without HygB or (B) As(III). Growth was monitored after 2–3 days at 30°C. (C) Sis1–GFP distribution was scored by fluorescence microscopy before and after exposure to 0.5 mM As(III). The fractions of cells containing 1–2 aggregates/cell and ≥3 aggregates/cell were determined by visual inspection of 102–131 cells per condition and time-point. Error bars represent s.d. from five independent biological replicates (*n*=5). Error bars on the top concern the total fraction of cells with aggregates, whilst those on the red bars concern the fraction of cells with ≥3 aggregates/cell. *P<0.05 compared with wild type (unpaired, two-tailed Student's *t*-test; blue, total fraction of cells with aggregates; red, ≥3 aggregates/cell). WT, wild-type cells. (D) Hsp104–GFP distribution was scored as in C in wild-type cells transformed with an empty plasmid (−) or with centromeric plasmids harboring *MSN2* or *MSN4*. (E) Cells were untreated or exposed to 0.75 mM As(III) for 1 h, and polysome profiles were obtained as described in C with three biologically independent replicates (*n*=3). A representative profile is shown. Right panel, the average P/M ratio is represented with s.d. *P<0.05 (unpaired, two-tailed Student's *t*-test).

We asked whether mutants encoding positive regulators of transcription that have reduced aggregation levels exhibit a general resistance to proteotoxic stress. Therefore, we tested growth of selected mutants lacking positive regulators of transcription (Msn4, Spt3, Srb2, Met18, Gat4, Ace2 and Spt8) in the presence of hygromycin B (HygB), a chemical that reduces translational fidelity leading to increased misincorporation of amino acids into nascent polypeptides causing them to misfold (Kim and Craig, 2005). The activator Msn4 identified in our screen has a paralog Msn2; Msn2 and Msn4 are partially redundant and they induce gene expression in response to several types of stress, including As(III) (Hosiner et al., 2009; Martínez-Pastor et al., 1996). We therefore included *msn2*Δ and the *msn2*Δ *msn4*Δ double mutant cells. Growth assays showed that *met18*Δ, *ace2*Δ and *msn2*Δ *msn4*Δ cells were HygB resistant (Fig. 5A). Hence, deletion of these positive regulators of transcription might mitigate proteotoxicity. Western blot analyses showed that all mutants had similar Hsp104 and HSP70 levels as wild-type cells after As(III) treatment (Fig. S4), suggesting that the reduced aggregation levels during As(III) stress observed in these mutants is not a result of high levels of molecular chaperones.

Msn2 and Msn4 were previously shown to be activated and to control induced expression of ∼60 genes during As(III) stress (Hosiner et al., 2009). Therefore, the anticipated effect of *MSN2/MSN4* deletion would be a decreased ability to cope with As(III) due to a compromised stress response. Instead, deletion of both genes resulted in As(III) resistance (Fig. 5B) (Hosiner et al., 2009), whereas overexpression of either *MSN2* or *MSN4* from a strong constitutive promoter caused sensitivity (Hosiner et al., 2009). To substantiate our data that suggest diminished proteotoxic stress in *msn2*Δ *msn4*Δ cells, we scored protein aggregation levels in cells carrying Sis1–GFP. Importantly, after 3 and 5 h of exposure the total fraction of cells with aggregates, as well as the fraction of cells with three or more Sis1-GFP foci/cell was clearly lower in *msn2*Δ *msn4*Δ compared to the wild type (Fig. 5C). In a reciprocal assay, moderate overexpression of *MSN2* or *MSN4* (native promoters; centromeric plasmids) in wild-type cells resulted in a higher fraction of cells with aggregates (Hsp104–GFP foci) than cells transformed with the empty vector after 1 h of exposure (Fig. 5D). A higher fraction of cells overexpressing *MSN4* also contained aggregates at the 3 h time-point compared to cells transformed with the empty vector. Hence, *MSN2*/*MSN4* gene dosage has a substantial impact on proteostasis. Interestingly, polysome-profiling assays revealed that the translational activity was similar for wild-type and *msn2*Δ *msn4*Δ cells in the absence of As(III) whereas *msn2*Δ *msn4*Δ cells were ∼2-fold more efficient than wild-type cells in reducing translation initiation during As(III) exposure (Fig. 5E). Thus, the ability of *msn2*Δ *msn4*Δ to efficiently decrease translation is likely responsible for diminished protein aggregation levels and for the HygB and As(III) resistance.

#### Negative regulators of transcription

**Fig. 6.**
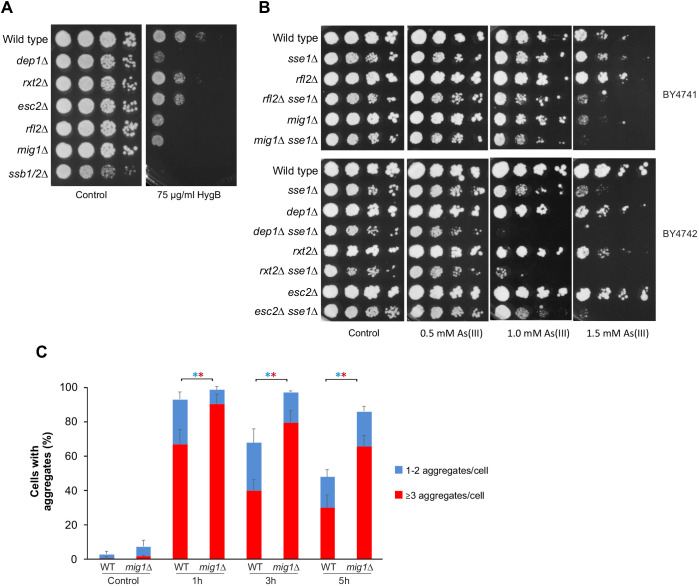
**Loss of negative regulators of transcription leads to enhanced protein aggregation.** (A) 10-fold serial dilutions of the indicated strains were plated onto YPD agar plates with or without HygB. Growth was monitored after 2–3 days at 30°C. (B) Loss of Sse1 exacerbates As(III) sensitivity of mutants that lack negative regulators of transcription. 10-fold serial dilutions of the indicated cells were plated onto YPD agar plates with or without As(III). Growth was monitored after 2–3 days at 30°C. (C) Sis1–GFP distribution was scored by fluorescence microscopy before and after exposure to 0.5 mM As(III). The fractions of cells containing 1–2 aggregates/cell and ≥3 aggregates/cell were determined by visual inspection of 111–161 cells per condition and time-point. Error bars represent s.d. from three independent biological replicates (*n*=3). Error bars on the top concern the total fraction of cells with aggregates; those on the red bars concern the fraction of cells with ≥3 aggregates/cell. *P<0.05 (unpaired, two-tailed Student's *t*-test; blue, total fraction of cells with aggregates; red, ≥3 aggregates/cell).

We next asked whether mutants encoding negative regulators of transcription that have enhanced aggregation levels exhibit sensitivity to proteotoxic stress. We selected a set of mutants lacking proteins that act as transcriptional repressors (Mig1), are components of the Rpd3L histone deacetylase complex (Dep1 and Rxt2) or are involved in chromatin silencing (Esc2 and Rlf2) and scored their growth in the presence of HygB. We included a mutant lacking the ribosome-associated chaperones Ssb1 and Ssb2 (*ssb1/2*Δ) as a positive control (Kim and Craig, 2005). All tested mutants were sensitive to HygB (Fig. 6A), suggesting that they might experience enhanced proteotoxic stress. To substantiate this, we took a genetic approach by introducing *SSE1* deletion into these mutants and testing whether this molecular chaperone is critical for their growth. Sse1 acts as a nucleotide exchange factor for HSP70 chaperones (Raviol et al., 2006; Shaner et al., 2006) and is required for Hsp104-dependent protein disaggregation (Kaimal et al., 2017), and cells lacking Sse1 have been shown to have enhanced protein aggregation levels (Koplin et al., 2010). There was a clear As(III)-dependent synthetic growth defect in all tested double mutants (Fig. 6B). Moreover, *dep1*Δ *sse1*Δ and *rxt2*Δ *sse1*Δ cells also grew poorly in the absence of As(III) and this growth defect was strongly aggravated in the presence of metalloid. These observations suggest that lack of these negative regulators of transcription sensitizes cells to protein folding stress. We confirmed enhanced protein aggregation levels in cells lacking *MIG1*, encoding a protein involved in glucose repression (Nehlin and Ronne, 1990); the total fraction of cells with aggregates as well as the fraction of cells with three or more Sis1–GFP foci/cell was significantly higher in *mig1*Δ than in the wild type at all time-points during exposure (Fig. 6C). Western blot analyses showed that all mutants had lower levels of Hsp104 compared to wild-type cells during As(III) exposure although some mutants (*esc2*Δ and *rfl2*Δ) had slightly elevated Hsp104 levels in the absence of stress (Fig. S5). HSP70 levels were similar in wild type and the mutants (Fig. S5). Hence, an inability to adjust chaperone levels might underlie the increase in protein aggregation observed in these mutants. Collectively, the above results indicate that loss of transcriptional control affects PQC through distinct mechanisms including translational control and protein folding.

### Translational repression is central for proteostasis and cell viability during As(III) stress

**Fig. 7.**
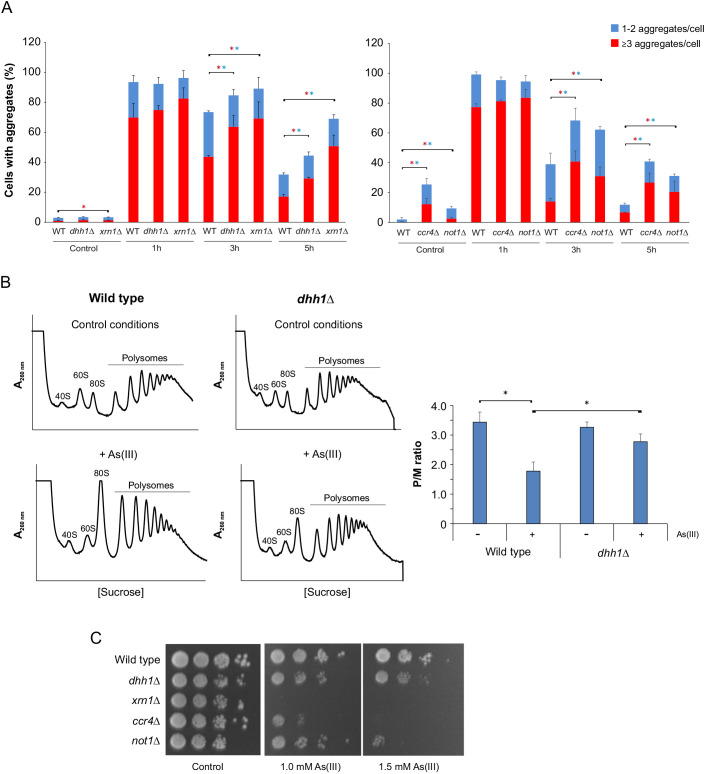
**Translational repression is central for proteostasis and cell viability during As(III) stress.** (A) Sis1–GFP distribution was scored by fluorescence microscopy before and after exposure to 0.5 mM As(III). The fractions of cells containing 1–2 aggregates/cell and ≥3 aggregates/cell were determined by visual inspection of 121–361 cells per condition and time-point. Error bars represent s.d. from three biological replicates (*n*=3). Error bars on the top concern the total fraction of cells with aggregates; those on the red bars concern the fraction of cells with ≥3 aggregates/cell. *P<0.05 (unpaired, two-tailed Student's *t*-test; blue, total fraction of cells with aggregates; red, ≥3 aggregates/cell). (B) Polysome profiles were obtained as described in Fig. 4C with three biologically independent replicates (*n*=3). A representative profile is shown. Right panel: the average P/M ratio is represented with s.d. *P<0.05 (unpaired, two-tailed Student's *t*-test). (C) 10-fold serial dilutions of the indicated strains were plated onto YPD agar plates with or without As(III). Growth was monitored after 2–3 days at 30°C. Images representative of three experiments.

The results above indicate that ongoing protein synthesis is detrimental during As(III) exposure. In particular, reduced translation was associated with less aggregates and improved growth for *rpb4*Δ and *msn2*Δ *msn4*Δ (Figs 4 and 5). Thus, translational repression might be important to control proteostasis during As(III) stress. To substantiate this, we measured protein aggregation levels in a selection of mutants lacking proteins with functions in translational repression and mRNA decay, including Dhh1, Xrn1, Not1 and Ccr4 (Coller and Parker, 2005; Holmes et al., 2004; Preissler et al., 2015). Indeed, all tested mutants had elevated protein aggregation levels during As(III) exposure; the total fraction of cells with Sis1–GFP foci as well as the fraction of cells with three or more Sis1–GFP foci/cell was significantly higher in *dhh1*Δ, *xrn1*Δ, *ccr4*Δ and *not1*Δ cells compared to wild-type cells at the 3 and 5 h time-points (Fig. 7A). Moreover, *xrn1*Δ, *ccr4*Δ and *not1*Δ cells had higher aggregation levels in the absence of stress. Cells lacking *DHH1* have been shown to be defective in translational repression during glucose starvation (Coller and Parker, 2005). Likewise, polysome profiling during As(III) exposure showed that *dhh1*Δ is ∼1.5-fold less efficient in inhibiting translation compared to wild-type cells (Fig. 7B). Growth assays indicated that cells lacking *DHH1*, *XRN1*, *CCR4* and *NOT1* are As(III) sensitive (Fig. 7C). Thus, the inability to efficiently repress translation is likely responsible for the enhanced protein aggregation levels and As(III) sensitivity observed in these mutants.

## DISCUSSION

### A comprehensive view of PQC during arsenite stress

Arsenic is a highly poisonous and carcinogenic metalloid that causes widespread protein misfolding and aggregation. Elucidating how protein aggregates are formed *in vivo*, the mechanisms by which they affect cells, and how cells prevent their accumulation is important for understanding the toxicity and pathogenicity of arsenic. Our genome-wide imaging screen uncovered novel genes and processes that may act specifically during As(III)-induced protein aggregation or as general proteotoxic stress factors. Several of the systems identified were known or anticipated to influence PQC, validating the screening approach. For example, a large set of hits with reduced levels of protein aggregation are associated with protein biosynthesis, supporting the notion that As(III) primarily triggers aggregation of nascent proteins. A set of hits with enhanced levels of protein aggregation are associated with protein folding, turnover and degradation. Thus, compromising the cellular capacity to correctly fold or degrade proteins increases the burden of misfolded and aggregated proteins during As(III) stress. We also identified genes and networks that were previously not linked to proteostasis, including proteins with functions related to signaling, chromatin organization and transcription. The mechanistic details through which many of the identified genes contribute to PQC remain to be established. Here, we have chosen to focus on selected hits and highlighted the importance of transcriptional and translational control to mitigate proteotoxicity.

### Protein aggregation is correlated with intracellular arsenic levels and As(III) toxicity

Genes related to arsenic transport and detoxification were present in both the enhanced and reduced aggregation sets. Loss of proteins that restrict cytosolic arsenic levels (e.g. Acr3, Yap8, Hog1, Ycf1 and Yap1) resulted in enhanced protein aggregation, whereas loss of proteins that contribute to arsenic influx (e.g. Fps1, Rgc1 and Rgc2, and Ptp2 and Ptp3) lead to fewer aggregates (Fig. S6). The *fps1*Δ, *rgc1*Δ *rgc2*Δ and *ptp2*Δ *ptp3*Δ mutants are As(III) resistant (Beese et al., 2009; Thorsen et al., 2006; Wysocki et al., 2001) whereas *acr3*Δ, *yap8*Δ, *ycf1*Δ, *yap1*Δ and *hog1*Δ are As(III) sensitive (Thorsen et al., 2006; Wysocki et al., 1997, 2004). These data suggest that restricting cellular accumulation of this metalloid is an important means to protect the proteome from As(III)-induced damage and toxicity. The finding that cells respond to As(III) by downregulating arsenic influx pathways (Jochem et al., 2019; Wysocki et al., 2001) and by increasing expression of arsenic export and sequestration systems (Talemi et al., 2014; Thorsen et al., 2007) supports this notion. Besides arsenic influx and efflux, additional mechanisms to mitigate proteotoxicity exist; selected mutants affected in transcriptional and translational control had a clear impact on As(III)-induced protein aggregation and toxicity with intracellular arsenic levels comparable to wild-type cells (Fig. S6).

Previous data have suggested that protein aggregation may contribute to As(III) toxicity. This was based on, among other data, the correlation of protein aggregation levels and sensitivity/resistance in a limited set of mutants (Jacobson et al., 2012). Our current study provides genome-wide support for this notion. We found strong correlations between enhanced protein aggregation and As(III) sensitivity, and between reduced aggregation and As(III) resistance (Fig. 2). Earlier studies indicated that proteins are the main target of As(III) during acute exposure (Talemi et al., 2014) and that hundreds of proteins aggregate (Ibstedt et al., 2014; Jacobson et al., 2012). Misfolded forms of these proteins might engage in extensive aberrant protein–protein interactions, resulting in an increased burden on PQC systems and an impact on cell viability (Ibstedt et al., 2014; Jacobson et al., 2012). Taken together, these findings support the view that protein aggregation contributes to As(III) toxicity and that aggregate management is crucial for cell survival. This mode of toxicity acts in parallel with previously described toxicity mechanisms, such as oxidative stress-induced damage to DNA, lipids and proteins, inhibition of DNA repair and disruption of enzyme function (Shen et al., 2013; Singh et al., 2011).

### Appropriate transcriptional control is important for proteostasis and As(III) resistance

We provide evidence that appropriate transcriptional control is crucial for PQC during As(III) exposure. First, a large number of transcription-related hits were found in both the enhanced and reduced aggregation sets (Tables S1, S2). Selected mutants lacking negative regulators of transcription accumulated more aggregates than wild-type cells and were As(III) sensitive. Genetic and biochemical data indicate that some of these mutants experience proteotoxic stress in the absence of As(III), and that protein aggregation is exacerbated during exposure. The molecular chaperone Sse1 was required to sustain growth of these mutants, supporting the notion that they suffer from proteotoxic stress (Fig. 6). Similarly, selected mutants lacking positive regulators of transcription were HygB resistant, suggesting that their absence might mitigate proteotoxicity (Fig. 5). Second, our data suggest that global transcription affects protein aggregation levels during As(III) stress. Inhibition of transcription with 1,10-phenantroline attenuated As(III)-induced aggregate formation (Fig. 4), which is similar to the effect observed with cycloheximide, a chemical inhibitor of translation (Jacobson et al., 2012). Moreover, protein aggregation levels were reduced in the *rpb4*Δ mutant, which has a decreased global transcription rate (Garrido-Godino et al., 2016). Thus, transcriptional inhibition by genetic or chemical means mitigates As(III)-induced protein aggregation. For some of the identified mutants, it is possible that altered transcription also affects global translation [i.e. by altering the number of nascent proteins synthesized that can be targeted by As(III) to misfold and aggregate]. This was the case for *rpb4*Δ; polysome-profiling assays demonstrated that *rpb4*Δ had a lower translational activity than the wild type in both the absence and presence of As(III) (Fig. 4). However, most of the transcription-related mutants identified here regulate expression of a limited set of target genes and are therefore expected to have little impact on global mRNA levels. Instead, these regulators are likely to affect aggregation through other mechanisms, for example by controlling gene targets that influence the cellular capacity to deal with intracellular As(III) and/or the damages caused by the metalloid. Indeed, some of the transcription-related mutants tested had lower levels of molecular chaperones (*dep1*Δ, *esc2*Δ, *rlf2*Δ, *rxt2*Δ and *mig1*Δ), accumulated high levels of intracellular arsenic (*yap1*Δ and *yap8*Δ) or had an altered capacity to control translation (*msn2*Δ *msn4*Δ). Interestingly, *msn2*Δ *msn4*Δ cells were resistant to As(III) and HygB, and showed reduced protein aggregation levels compared to wild-type cells (Fig. 5). This finding was unexpected since Msn2 and Msn4 regulate As(III)-induced expression of ∼60 genes (Hosiner et al., 2009). It was previously hypothesized that a chronic activation of general stress factors by Msn2 and Msn4 may contribute to As(III) sensitivity (Hosiner et al., 2009). Here, we demonstrate that the absence of Msn2 and Msn4 promotes strong translation inhibition in response to As(III) (Fig. 5). Notably, translation activity is similar in wild type and *msn2*Δ *msn4*Δ prior to stress, and *MSN2*/*MSN4* deletion does not affect Hsp104 and Hsp70 protein levels (Fig. S4) or intracellular arsenic accumulation (Fig. S6). Hence, *msn2*Δ *msn4*Δ cells are not pre-adapted to stress by increased folding capacity and/or lower translation activity but respond to As(III) by robust translation inhibition. This capacity to efficiently inhibit translation is likely responsible for the diminished aggregation levels observed in *msn2*Δ *msn4*Δ cells, as well as for their HygB and As(III) resistance. The targets of Msn2/Msn4 that regulate translation inhibition remain to be identified. Previous studies have shown that As(III)-exposed cells downregulate gene expression associated with protein synthesis and upregulate expression of genes related to protein folding and degradation (Haugen et al., 2004; Ibstedt et al., 2014; Thorsen et al., 2007). Moreover, yeast cells decrease expression of aggregation-prone proteins during As(III) exposure (Ibstedt et al., 2014), possibly as a means to mitigate aggregation and toxicity. Collectively, these data support the notion that accurate transcriptional control is crucial in protecting cells from the effects of accumulated misfolded and aggregated proteins. The mechanisms by which transcriptional regulators impact PQC appear to be distinct.

### Translational control is crucial to mitigate As(III)-induced proteotoxicity

Our data highlights that translational control is crucial for proteostasis and As(III) resistance. First, a large proportion of the mutants with reduced aggregation (∼25% of the hits) are part of highly connected networks involved in cytoplasmic translation (e.g. ribosomal proteins), rRNA modification and processing, and ribosome assembly (Fig. 2; Fig. S2). These findings are in line with previous studies showing that proteins are particularly susceptible to As(III)-induced aggregation during translation and folding (Ibstedt et al., 2014; Jacobson et al., 2012), and that proteins with high translation rates are predominantly at risk (Ibstedt et al., 2014; Weids et al., 2016). Second, we demonstrate that diminished translation mitigates As(III)-induced protein aggregation and toxicity. Polysome-profiling assays showed that wild-type cells repress translation during As(III) exposure (Fig. 4). This is likely a result of increased eIF2α phosphorylation (Guerra-Moreno et al., 2015), inhibition of translation initiation (Liu et al., 2013) and reduction of ribosomal protein levels (Guerra-Moreno et al., 2015). Importantly, translation repression appears to be crucial for safeguarding the proteome from As(III)-induced damage. Low translational activity of *rpb4*Δ protected this mutant from As(III)-induced protein aggregation and toxicity (Fig. 4). Similarly, an improved efficiency in translational repression, as shown for *msn2*Δ *msn4*Δ cells, mitigated protein aggregation and resulted in As(III) resistance (Fig. 5). Our data also emphasize the key role of translation initiation control in response to As(III) in contrast to other proteotoxic stresses, like heat shock (Fig. 4E). Third, we demonstrate that mutants lacking proteins with functions in translational repression have enhanced protein aggregation levels and exhibit As(III) sensitivity (Fig. 7). These mutants accumulated comparable intracellular arsenic to that of wild-type cells (Fig. S6). Thus, low translation and efficient translational repression is beneficial during As(III) exposure whilst lack of translational repression is detrimental for the cell. Together, these data strongly support the notion that appropriate control at the translational level is crucial to mitigate As(III)-induced proteotoxicity.

### Implications for human disease processes

Previously, we found several homologs of yeast proteins that aggregated during As(III) exposure to be present in human disease-associated aggregates in AD, PD and amyotrophic lateral sclerosis (ALS), suggesting that the mechanisms underlying protein aggregation in stress-exposed yeast cells may be relevant to human disease processes (Ibstedt et al., 2014; Weids et al., 2016). The hit list from the current screen is also interesting in the light of human pathogenesis as several homologs to proteins related to human diseases were identified (Tables S1, S2). For example, lack of Tdp1, encoding tyrosyl-DNA phosphodiesterase I, resulted in increased protein aggregation levels. Mutation in the human homolog, TDP1, results in the inherited neurodegenerative disorder SCAN1 (Takashima et al., 2002). Thus, SCAN1 patients may be susceptible to conditions of proteotoxic stress, such as chronic exposure to low levels of metals. Similarly, lack of Ltn1 resulted in elevated aggregation levels during As(III) exposure. Ltn1 is the yeast homolog of mammalian listerin (LTN1), an E3 ubiquitin ligase implicated in neurodegeneration (Chu et al., 2009). The screen also identified several components of the elongator complex (ELP1–ELP6), which is primarily involved in tRNA modifications (Johansson et al., 2018); deletion of *ELP1* (also known as *IKI3*), *ELP2*, *3*, *4* or *6* caused reduced levels of protein aggregates during As(III) exposure. Mutations in the human Elp1 homolog IKAP (also known as ELP1) causes familial dysautonomia, a rare neurodegenerative disease that is associated with growth abnormalities and degradation of sensory functions, and patients suffering from this disease show reduced levels of the wobble uridine tRNA modification (Dalwadi and Yip, 2018). Elp3 is suggested to be a modifier of ALS, a fatal degenerative motor neuron disorder, indicating a possible link between tRNA modifications and neurodegeneration (Bento-Abreu et al., 2018). Thus, members of the elongator complex are implicated in disease processes, as well as PQC during metalloid exposure. Chronic heavy metal and metalloid exposure may promote the progression of certain neurodegenerative and age-related disorders, such as AD and PD (Caudle et al., 2012; Chin-Chan et al., 2015; Gong and O'Bryant, 2010; Wang and Du, 2013); however, the underlying mechanisms remain largely unknown. A better understanding of the role of the proteins above in PQC may shed novel light on disease processes associated with metal exposure.

### Conclusions

Collectively, this work provides a comprehensive view of cellular machineries that impinge on proteostasis during As(III) stress and highlights the importance of transcriptional and translational control. The broad network of cellular systems identified here provides a valuable resource and a framework for dedicated follow-up studies of the molecular underpinnings of arsenic toxicity and pathogenesis.

## MATERIALS AND METHODS

### Yeast strains and culturing conditions

*S. cerevisiae* strains used in this study are listed in Table S4. Most strains are based on BY4741 and BY4742 (Brachmann et al., 1998), W303-1A (Thomas and Rothstein, 1989) and the yeast deletion collection (Giaever et al., 2002). Double mutants were generated by crossing haploid single mutants using standard procedures and all double mutants were confirmed by PCR. Yeast cells were grown in minimal synthetic complete (SC) medium (0.67% yeast nitrogen base) supplemented with auxotrophic requirements and 2% glucose as a carbon source or in rich yeast peptone dextrose (YPD) medium. Growth assays on solid agar were carried out as previously described (Wysocki et al., 2004). Sodium arsenite (NaAsO_2_), 1,10-phenantroline (both from Sigma-Aldrich) and hygromycin B (Formedium) were added to the cultures at the indicated concentrations.

### Yeast library creation and automated high-content microscopy

The yeast deletion library harboring Hsp104–GFP was created as previously described (Babazadeh et al., 2019) by crossing the Hsp104–GFP query strain into a genome-wide collection of viable yeast single deletion mutants (SGA-v2, Boone laboratory, University of Toronto, Canada) using a synthetic genetic array approach (Tong et al., 2001, 2004). Cells from the library were transferred into 96-well plates using a liquid handling robot (Microlab STAR, Hamilton Company), and treated with 0.25 mM As(III) for 2 h followed by fixation with 3.7% formaldehyde. Then cells were washed twice in 1× phosphate-buffered saline (PBS) and transferred into 96-well glass bottom plates for imaging. Automatic image acquisition was performed using a high-content microscope (ImageXpress MICRO, Molecular Devices); 25 images were acquired for each mutant. Quantification of number of cells with aggregates and number of aggregates per cell was performed using MetaXpress (version 3, Molecular Devices) software.

### Bioinformatics and network analyses

Categories of over-represented protein functions in our datasets were identified using FunCat at Munich Information Center for Protein Sequences (MIPS) (http://mips.gsf.de). Enriched functional categories were set with a false discovery rate (FDR) of <0.05 using the Benjamini–Hochberg procedure (Benjamini and Hochberg, 1995). Protein–protein interaction networks were constructed using the STRING database (string-db.org/) (Snel et al., 2000; Szklarczyk et al., 2017) with the organism set to *Saccharomyces cerevisiae* and the confidence score to the highest (0.9). The functional diversity of the gene lists was visualized by mapping each hit onto the global yeast genetic interaction network using CellMap (Costanzo et al., 2016; Usaj et al., 2017).

### Fluorescence microscopy

Yeast cells expressing Hsp104–GFP (Table S4) or Sis1–GFP (Malinovska et al., 2012) fusion proteins were grown to mid-log phase in SC medium and treated with 0.5 mM As(III). Where indicated, 0.1 mg/ml 1,10-phenantroline was added. At the indicated time-points, cell samples were fixed with formaldehyde for 30 min at room temperature and washed with PBS. The GFP signals were observed using a Zeiss Axiovert 200 M (Carl Zeiss MicroImaging) fluorescence microscope equipped with Plan-Apochromat 1.40 objectives and appropriate fluorescence light filter sets. Images were taken with a digital camera (AxioCamMR3) and processed with Zeiss Zen software. To quantify protein aggregation, the total fraction of cells with aggregates (Hsp104–GFP or Sis1-GFP foci) as well as the fraction of cells having three or more aggregates/cell was determined using Image J software.

### Transcriptional errors

Transcription error assays were performed largely as described previously (Irvin et al., 2014). Patches of cells were grown on YPD medium overnight at 30°C, replica plated onto SC medium lacking histidine (His^−^) either with or without As(III) and incubated at 30°C for 3 days. Quantitative assays were performed in liquid medium. For this, cells were grown in YPD at 30°C overnight and then plated onto His^−^ plates either with or without As(III) as well as onto YPD plates to quantify the mean frequency of His^+^ cells relative to total number of viable cells. Plates were incubated at 30°C for 3 days and individual colonies were scored.

### Arsenic uptake

Intracellular arsenic was measured as described previously (Thorsen et al., 2006). Briefly, exponentially growing cells were exposed to As(III) for 1 h, collected and washed twice in ice-cold water. The cell pellet was then resuspended in water, boiled for 10 min, and centrifuged (17,000 ***g*** for 10 min) to collect the supernatant. The arsenic content of each sample was measured using a flame atomic absorption spectrometer (3300, Perkin Elmer) or by inductively coupled plasma-mass spectrometry (ICP-MS) using an ICAP Q ICP-MS (Thermo Fisher Scientific) with an SC-FAST automated sample introduction system (Elemental Scientific). Prior to analysis by ICP-MS, the samples were diluted 20 times with ultrapure water from a GenPure water purification system (Thermo Scientific Barnstead, resistivity 18.2 MΩ cm) and acidified to 1% volume HNO_3_ (Sharlau, HNO_3_, 65% for trace analysis). The instrument was operated in the Kinetic Energy Discrimination (KED) mode with helium as the collision gas to remove potential interference from ArCl^+^ at *m*/*z*=75. Calibration was performed using a set of arsenic standards with concentrations up to 500 µg l^−1^. A solution of 1 µg l^−1^ indium was continuously injected for internal standardization. The detection limit is estimated to 0.1 µg As l^−1^.

### Protein analyses

Cells were grown in SC medium until log phase and exposed to 0.5 mM As(III) during 1 h. A volume of cells corresponding to OD_600_=1 was collected before and after As(III) exposure, treated with 2 M lithium acetate and incubated on ice for 5 min, centrifuged (17,000 ***g*** for 2 min) and the supernatant was discarded. A second treatment with 0.4 M NaOH was performed, followed by a 5 min incubation on ice. After centrifugation (17,000 ***g*** for 2 min), cells were resuspended in 2× SDS loading buffer (125 mM Tris-HCl pH 6.7, 6% SDS, 2% glycerol, 10% β-mercaptoethanol and Bromophenol Blue) and boiled for 5 min at 95°C. Protein samples were loaded on 10% TGX Stain-Free Gels and run at 120 V. Protein was transferred to a PVDF membrane using a Trans-Blot Turbo transfer system (Bio-Rad, Hercules, CA, USA), according to the manufacturer's protocol. Membranes were blocked with 5% Blotting Grade Blocker (Bio-Rad) in Tris-buffered saline containing 0.05% Tween 20 (5% non-fat dry milk/TBS-T) (w/v), followed by an overnight incubation with the primary antibodies: anti-Hsp104 (1:5000; rabbit, ab69549, Abcam, Cambridge, UK) or anti-Hsp70 (1:2500; mouse, ab47455, Abcam). The following day, antibodies were washed three times with TBS-T, followed by a 1.5 h incubation with the respective secondary antibodies: StarBright700 anti-rabbit-IgG (10000068187, 1:5000) or StarBright700 anti-mouse-IgG (10000068185, 1:2500) both from Bio-Rad (Hercules, CA, USA). Thereafter, four washes with TBS-T were performed and the signal was detected using ChemiDoc (Bio-Rad, Hercules, CA, USA). The same protocol was performed for detection of the loading control, using the primary antibody anti-Pgk1 (1:5000; mouse, ab113687, Abcam, Cambridge, UK) and secondary antibody DyLight650 anti-mouse-IgG (84545, Invitrogen, Waltham, Massachusetts, USA). Images recovered from ChemiDoc were treated using the Image Lab Software (Bio-Rad, Hercules, CA, USA).

### Polysome-profiling assays

To perform polyribosome profile analyses, a volume of an exponential growing cell culture (OD_600_ of 0.6–1.0) corresponding to 50 OD_600_ units was collected. Then, cycloheximide (10 mg/ml) was added to a final concentration of 0.1 mg/ml and incubated 5 min on ice with occasional mixing. After centrifugation at 1968 ***g*** for 5 min at 4°C, cells were resuspended and washed twice in 2 ml of cold lysis buffer (20 mM Tris-HCl pH 8.0, 140 mM KCl, 5 mM MgCl₂, 0.5 mM DTT, 0.1 mg/ml cycloheximide, 0.5 mg/ml heparin, 1% Triton X-100). Then, cells were resuspended in 700 µl lysis buffer and transferred into a 2 ml screw-cap tube containing 500 µl of glass beads. Cells were broken by vortexing eight times during 30 s, with 30 s of incubation on ice in between. After centrifugation (3075 ***g***, 5 min, 4°C), the supernatant was transferred into a new tube and centrifuged again (7871 ***g***, 5 min, 4°C). RNA from the supernatant was quantified. Glycerol was added to a final concentration of 5% and samples were snap frozen and stored at −80°C. Samples were loaded onto 10–50% sucrose gradients and separated by ultracentrifugation for 2 h and 40 min at 35,000 rpm in a Beckman SW41Ti rotor at 4°C. The ultraviolet detection at absorbance (A)_260nm_ generated the general polyribosome profiles using Density Gradient Fractionation System and Isco UA-6 ultraviolet detector (Teledyne Isco, Lincoln, NE, USA).

## Supplementary Material

Supplementary information

Reviewer comments
