# Supplementary material for: Genome-wide imaging screen uncovers molecular determinants of arsenite-induced protein aggregation and toxicity
Source: J Cell Sci. 2021 Jun 4;134(11):jcs258338. doi: 10.1242/jcs.258338 (PMC8214759; doi:10.1242/jcs.258338)
Supplement: Supplementary information [file joces-134-258338-s1.pdf]

S1A

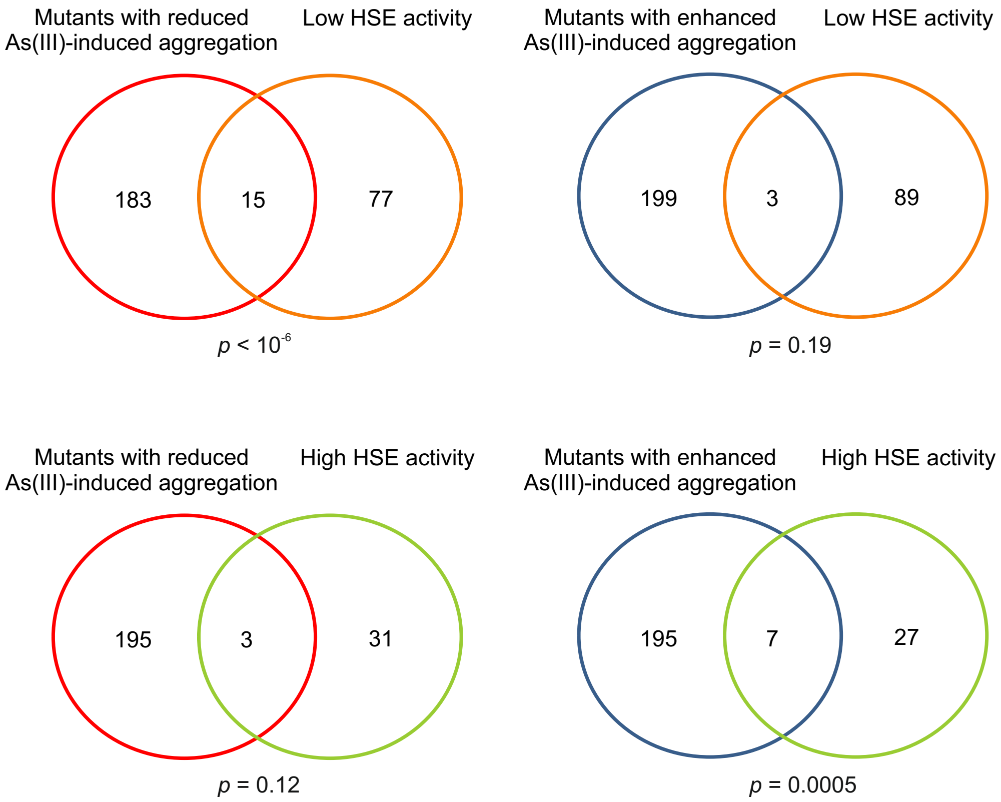

S1B

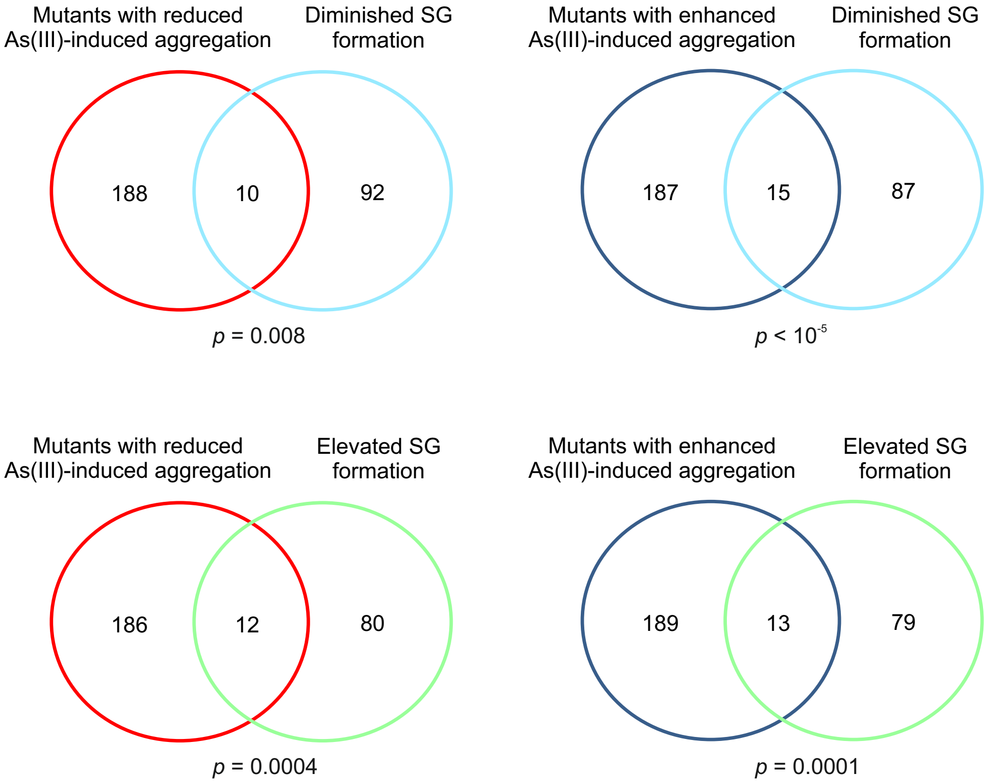

Journal of Cell Science • Supplementary information

S1C

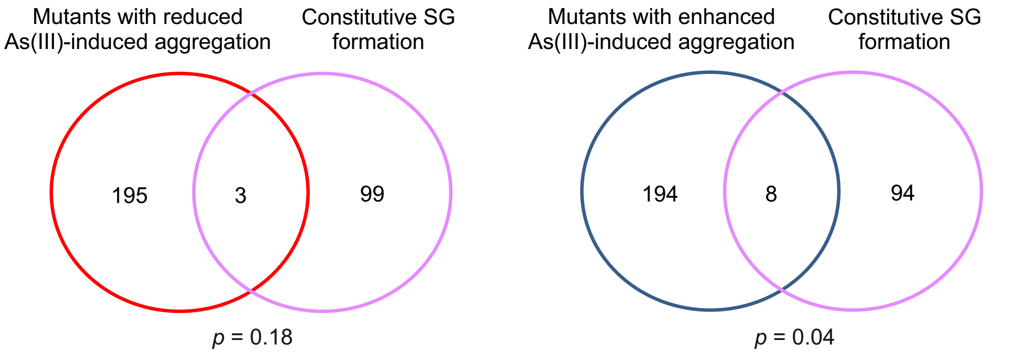
Mutants with reduced
As(III)-induced aggregation
Constitutive SG
formation
195
3
99
p = 0.18
Mutants with enhanced
As(III)-induced aggregation
Constitutive SG
formation
194
8
94
p = 0.04

S1D

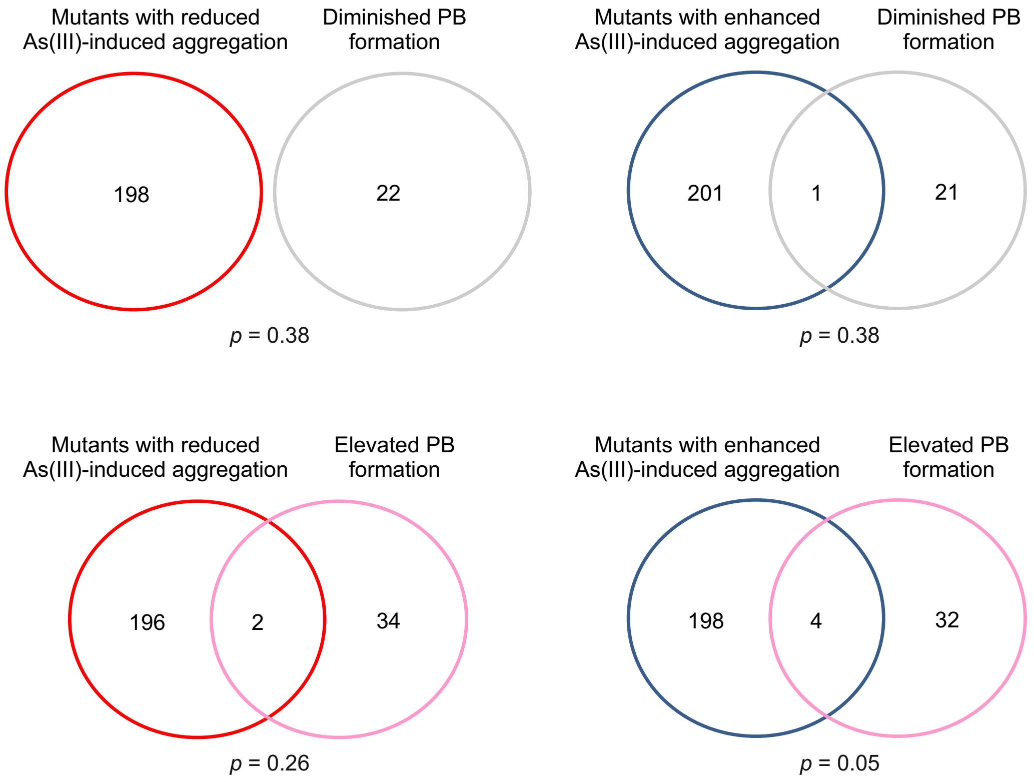
Mutants with reduced
As(III)-induced aggregation
Diminished PB
formation
198
22
p = 0.38
Mutants with enhanced
As(III)-induced aggregation
Diminished PB
formation
201
1
21
p = 0.38
Mutants with reduced
As(III)-induced aggregation
Elevated PB
formation
196
2
34
p = 0.26
Mutants with enhanced
As(III)-induced aggregation
Elevated PB
formation
198
4
32
p = 0.05

S1E

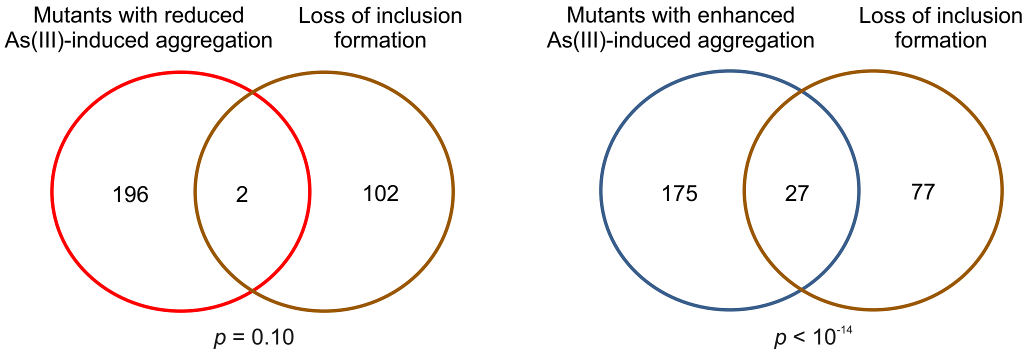
Mutants with reduced
As(III)-induced aggregation
Loss of inclusion
formation
196
2
102
p = 0.10
Mutants with enhanced
As(III)-induced aggregation
Loss of inclusion
formation
175
27
77
p < 10^-14

S1F

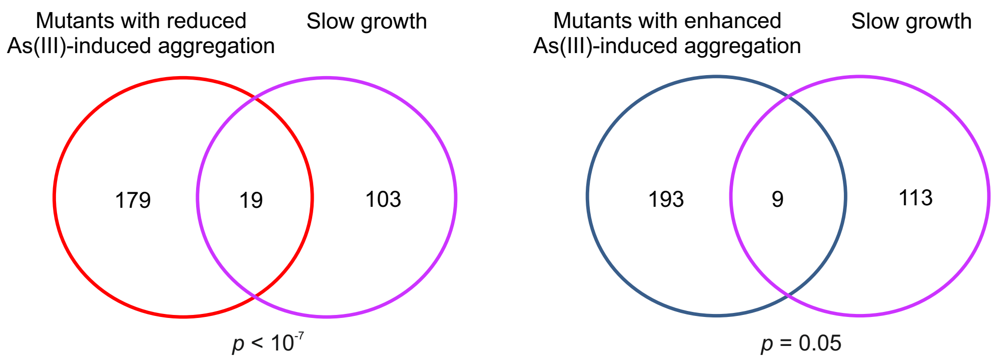

Figure S1. Correlation between protein aggregation in the presence of As(III) and other genome-wide data-sets.
(A) Correlation between protein aggregation in the presence of As(III) and HSE (Heat Shock Element) reporter activity. The Venn diagrams show the overlap between mutants with reduced or enhanced levels of protein aggregation and mutants having either high or low HSE activity (Brandman et al., 2012).
(B,C) Correlation between protein aggregation in the presence of As(III) and SG (stress granule) formation. The Venn diagrams show the overlap between mutants with reduced or enhanced levels of protein aggregation and mutants having high or low SG formation **(B)** or constitutive SG formation **(C)** (Buchan et al., 2013; Yang et al., 2014).
(D) Correlation between protein aggregation in the presence of As(III) and PB (Processing Body) formation. The Venn diagrams show the overlap between mutants with reduced or enhanced levels of protein aggregation and mutants with either elevated or diminished PB formation (Buchan et al., 2013).
(E) Correlation between protein aggregation in the presence of As(III) and mutants with defects in assembly of large inclusions during heat stress. The Venn diagrams show the overlap between mutants with reduced or enhanced levels of protein aggregation and of mutants with defects in assembly of large inclusions during heat stress (Babazadeh et al., 2019).
(F) Correlation between protein aggregation in the presence of As(III) and slow growth in minimal synthetic complete growth medium. The Venn diagrams show the overlap between mutants with reduced or enhanced levels of protein aggregation and mutants that grow slowly (Zackrisson et al., 2016).
Significance of the overlaps was calculated by the hyper-geometric test and the corresponding *p*-values are indicated.

Journal of Cell Science • Supplementary information

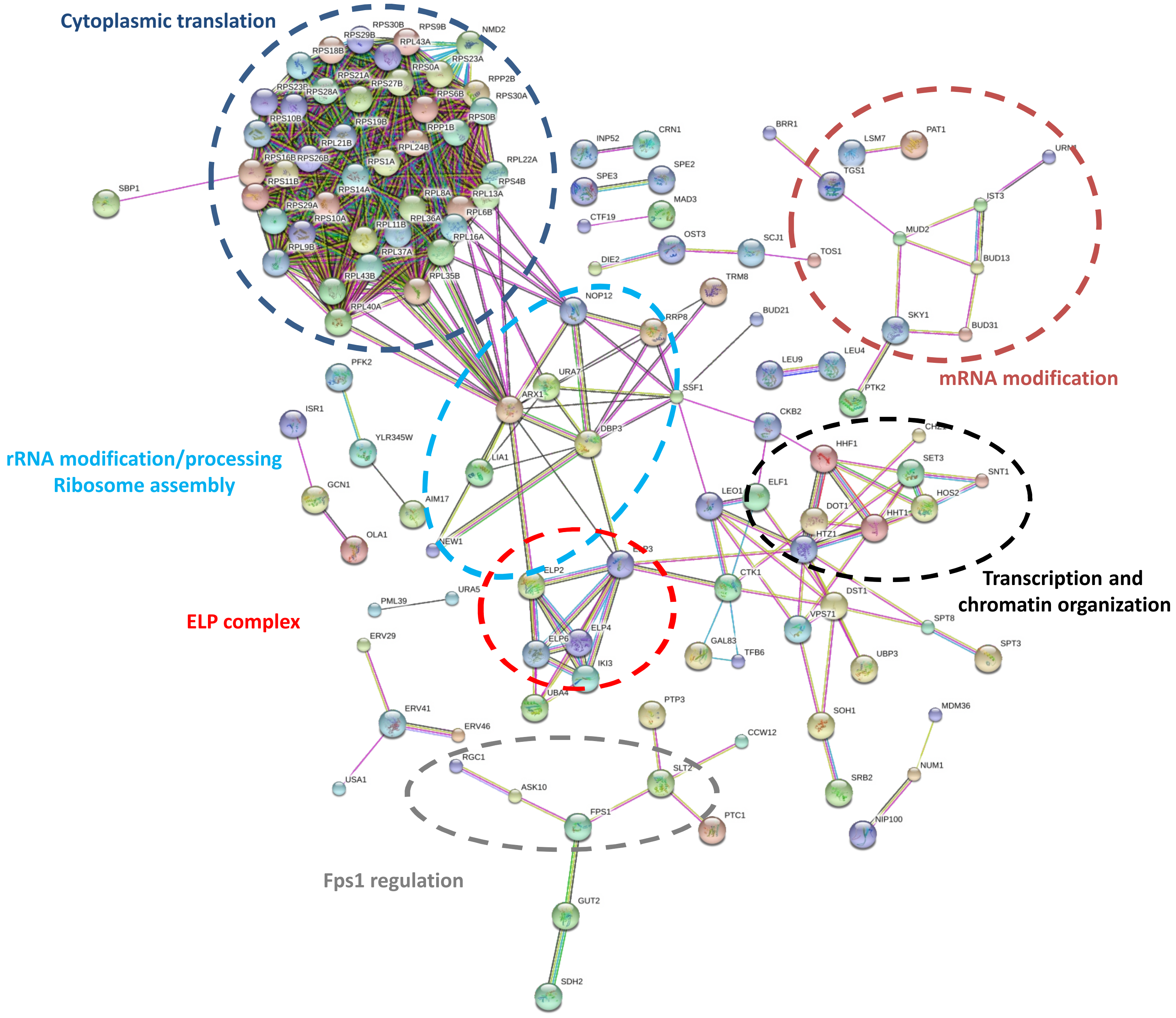

Figure S2. Protein-protein interaction networks among hits with reduced aggregation levels. Protein-protein interaction networks were constructed and visualized using the STRING database (Snel et al., 2000; Szklarczyk et al., 2017) (evidence view, confidence score set to 0.9 (highest confidence). Only connected proteins are shown.

Journal of Cell Science • Supplementary information

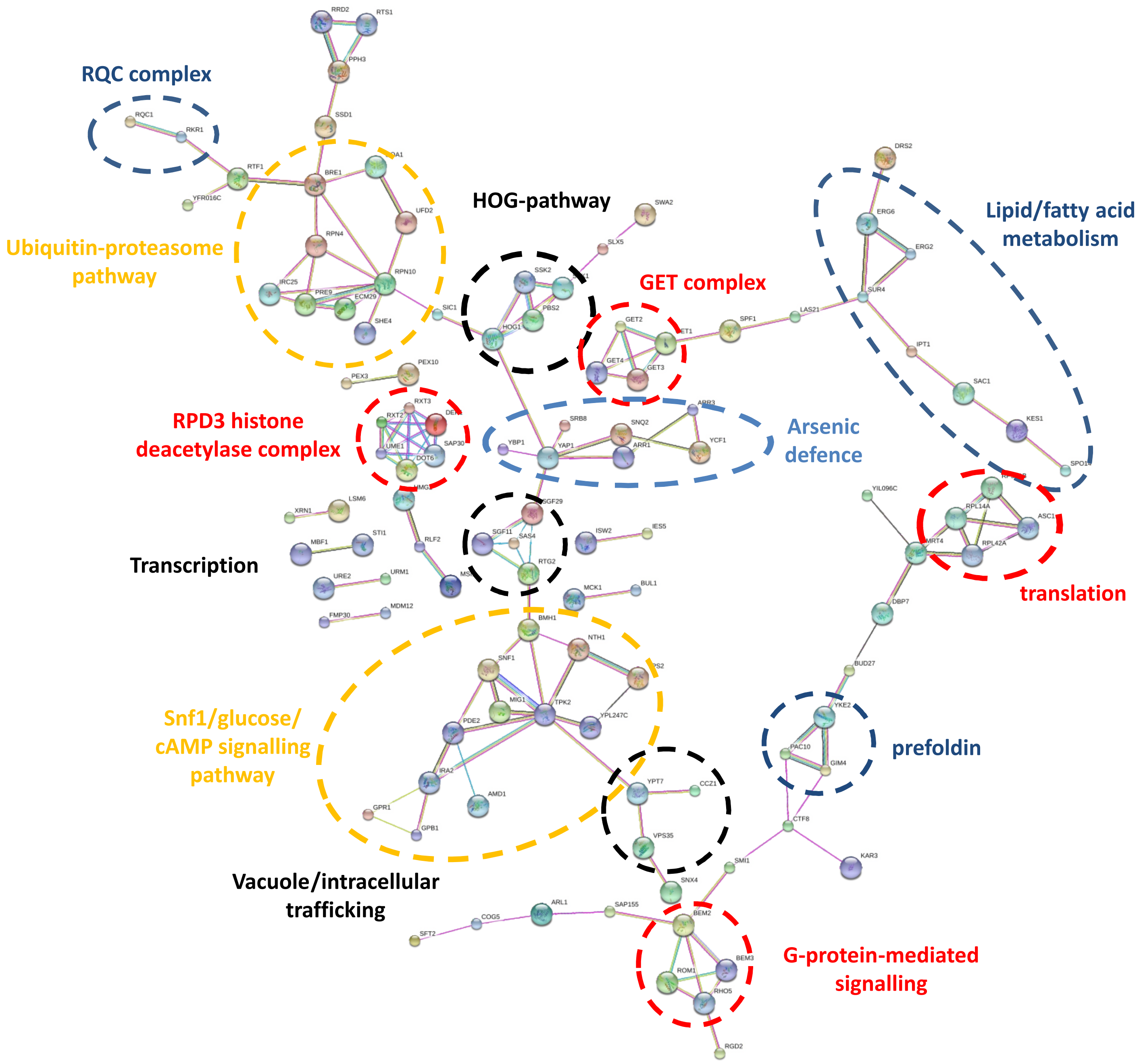

Figure S3. Protein-protein interaction networks among hits with enhanced aggregation levels. Protein-protein interaction networks were constructed and visualized using the STRING database (Snel et al., 2000; Szklarczyk et al., 2017) (evidence view, confidence score set to 0.9 (highest confidence). Only connected proteins are shown.

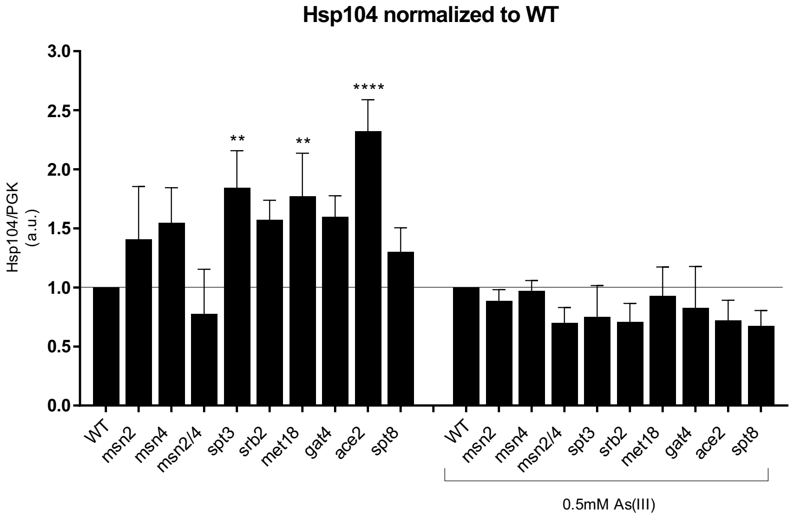

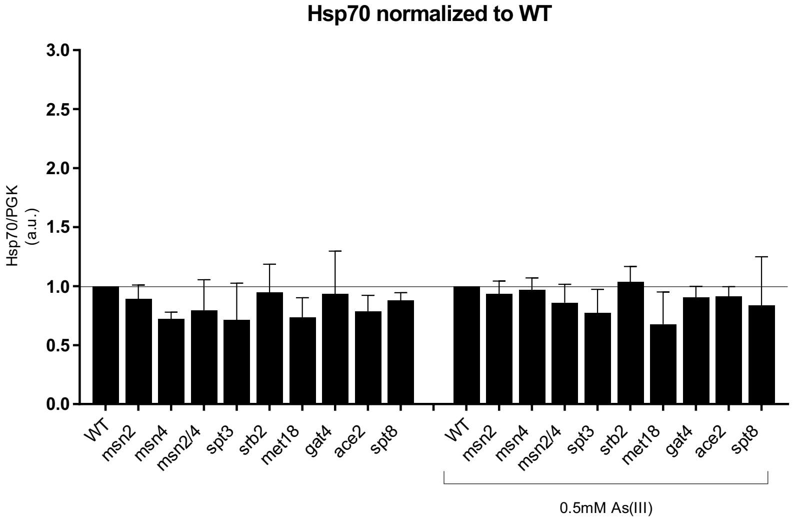

Figure S4. Hsp104 and Hsp70 protein levels in wild type cells and deletion mutants lacking positive regulators of transcription. Hsp104 and Hsp70 protein levels in wild type cells and deletion mutants lacking positive regulators of transcription before and after 1 h of 0.5 mM As(III) treatment. Hsp104 and Hsp70 levels were normalized to the levels of the loading control Pgk1. Protein levels were normalized to the wild type indicated by the horizontal line. Error bars represent standard deviations (S.D.) from four independent biological replicates (n=4). Statistical analyses were performed by One-Way ANOVA with Dunnett Test (***p*<0.005, *****p*<0.00005)

Journal of Cell Science • Supplementary information

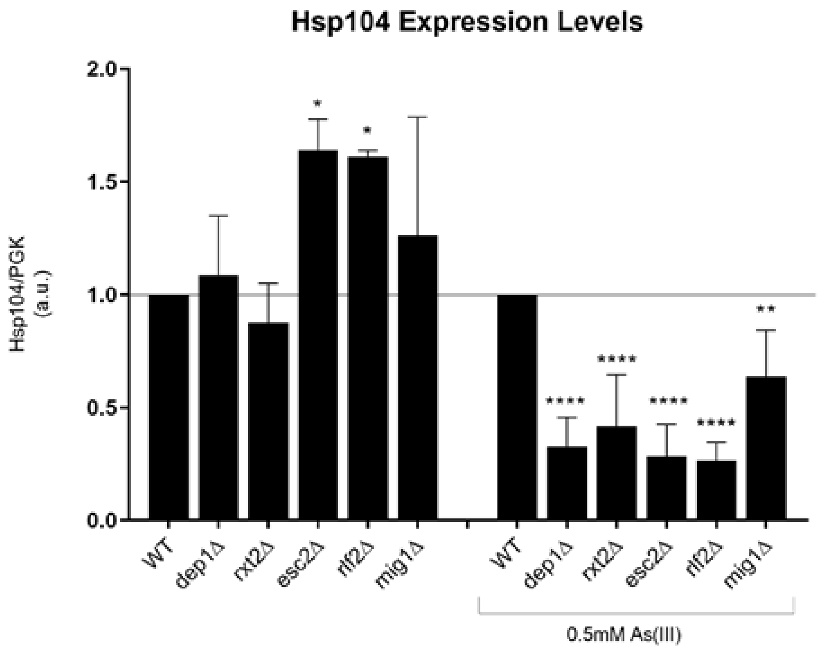

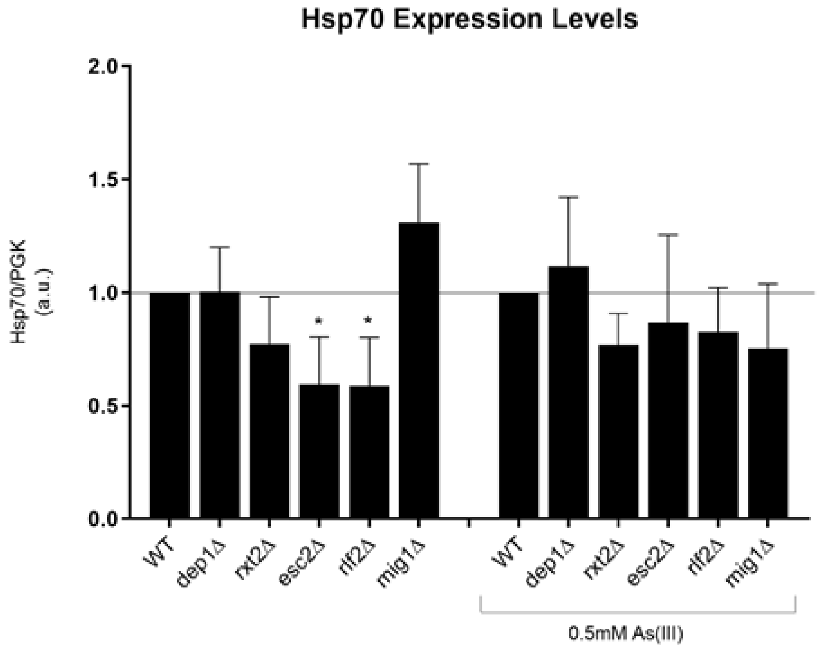

Figure S5. Hsp104 and Hsp70 protein levels in wild type cells and deletion mutants lacking negative regulators of transcription. Hsp104 and Hsp70 protein levels in wild type cells and deletion mutants lacking negative regulators of transcription before and after 1 h of 0.5 mM As(III) treatment. Hsp104 and Hsp70 levels were normalized to the levels of the loading control Pgk1. Protein levels were normalized to the wild type indicated by the horizontal line. Error bars represent standard deviations (S.D.) from six independent biological replicates (n=6). Statistical analyses were performed by One-Way ANOVA with Dunnett Test (***p*<0.005, *****p*<0.00005)

Journal of Cell Science • Supplementary information

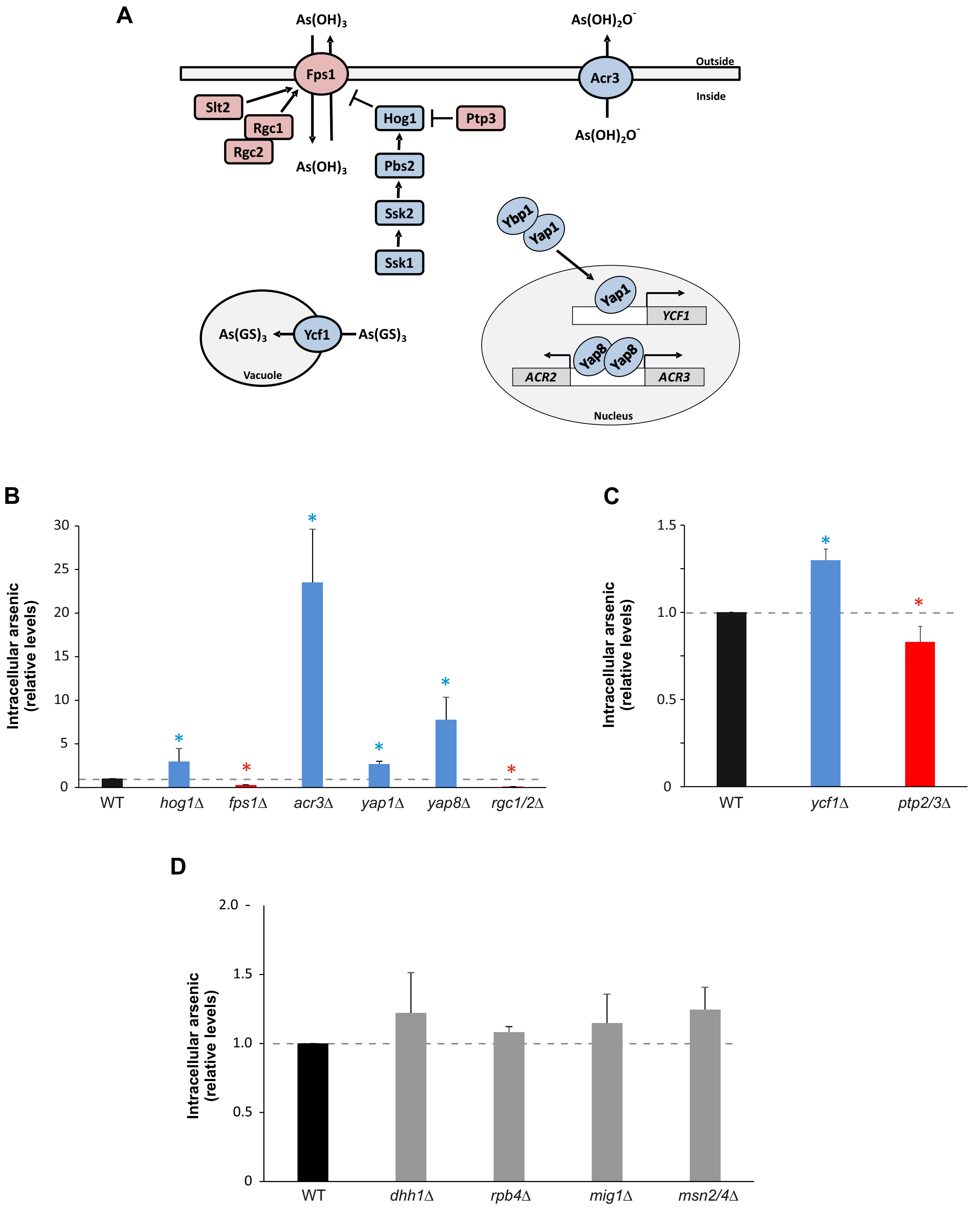

Figure S6. Correlation intracellular arsenic and protein aggregation. (A) Cartoon showing proteins that regulate arsenic transport and intracellular arsenic levels. Loss of proteins coloured red reduces protein aggregation whilst loss of blue-coloured proteins enhances protein aggregation levels during As(III) exposure. **(B,C,D)** Intracellular arsenic. Cells were exposed to As(III) for 1 h, and intracellular arsenic levels were determined. The data shown represents the average of at least two independent biological replicates (n=2) and the error bars represent the S.D. Red bars represent mutants with less intracellular arsenic whilst blue bars represent mutants with more intracellular arsenic compared to wild type cells. Grey bars represent mutants with similar intracellular arsenic as the wild type. Relative values are shown to enable comparison between strain backgrounds. Statistical analyses were performed by Student's t-test and significant differences are indicated by * (*p*<0.05).

Journal of Cell Science • Supplementary information

Supplemental Tables

Table S1. Hits mutants with reduced aggregation

Click here to download Table S1

Table S2. Hits mutants with enhanced aggregation

Click here to download Table S2

Table S3. Datasets comparisons

Click here to download Table S3

Table S4. Yeast strains

Click here to download Table S4
