# Supplementary material for: Genome-wide imaging screen uncovers molecular determinants of arsenite-induced protein aggregation and toxicity
Source: J Cell Sci. 2021 Jun 4;134(11):jcs258338. doi: 10.1242/jcs.258338 (PMC8214759; doi:10.1242/jcs.258338)
Supplement: Reviewer comments [file joces-134-258338_review_history.pdf]

Genome-wide imaging screen uncovers molecular determinants of arsenite-induced protein aggregation and toxicity

Stefanie Andersson, Antonia Romero, Joana Isabel Rodrigues, Sansan Hua, Xinxin Hao, Therese Jacobson, Vivien Karl, Nathalie Becker, Arghavan Ashouri, Sebastien Rauch, Thomas Nyström, Beidong Liu and Markus J. Tamás
DOI: 10.1242/jcs.258338

Editor: John Heath

Review timeline

Original submission:	21 December 2020
Editorial decision:	1 February 2021
First revision received:	21 April 2021
Accepted:	3 May 2021

Original submission

First decision letter

MS ID#: JOCES/2020/258338

MS TITLE: Genome-wide imaging screen uncovers molecular determinants of arsenite-induced protein aggregation and toxicity

AUTHORS: Stefanie Andersson, Antonia Romero, Joana Isabel Rodrigues, Sansan Hua, Xinxin Hao, Therese Jacobson, Vivien Karl, Nathalie Becker, Arghavan Ashouri, Thomas Nystrom, Beidong Liu, and Markus J Tamas
ARTICLE TYPE: Research Article

We have now reached a decision on the above manuscript.

To see the reviewers' reports and a copy of this decision letter, please go to: https://submit-jcs.biologists.org and click on the 'Manuscripts with Decisions' queue in the Author Area. (Corresponding author only has access to reviews.)

As you will see, the reviewers raise a number of substantial criticisms that prevent me from accepting the paper at this stage. They suggest, however, that a revised version might prove acceptable, if you can address their concerns. If you think that you can deal satisfactorily with the criticisms on revision, I would be pleased to see a revised manuscript. We would then return it to the reviewers.

We are aware that you may be experiencing disruption to the normal running of your lab that makes experimental revisions challenging. If it would be helpful, we encourage you to contact us to discuss your revision in greater detail. Please send us a point-by-point response indicating where you are able to address concerns raised (either experimentally or by changes to the text) and where you will not be able to do so within the normal timeframe of a revision. We will then provide further guidance. Please also note that we are happy to extend revision timeframes as necessary.

Please ensure that you clearly highlight all changes made in the revised manuscript. Please avoid using 'Tracked changes' in Word files as these are lost in PDF conversion.

© 2021. Published by The Company of Biologists under the terms of the Creative Commons Attribution License (https://creativecommons.org/licenses/by/4.0/).

I should be grateful if you would also provide a point-by-point response detailing how you have dealt with the points raised by the reviewers in the 'Response to Reviewers' box. Please attend to all of the reviewers' comments. If you do not agree with any of their criticisms or suggestions please explain clearly why this is so.

Reviewer 1

Advance summary and potential significance to field

The paper by Andersson et al describes a screen for genes involved, either directly or indirectly, in protein aggregation by arsenite in yeast. The methods used are appropriate and controls were properly introduced. Although other screens with similar purposes have been already published (see below), the present work focuses on a specific stressor that had not been characterized at a genome-wide level and constitutes a valuable study. In my view, however, there are some major issues that should be addressed before publication is considered.

Comments for the author

Major points:
1. Hsp104 co-localizes with Pab1 in SGs after heat shock (Cherkasov et al 2013).
Since arsenite also induces SG formation, the co-localization extent of Hsp104 with SG reporter proteins under the specific conditions used by the authors should be shown.
2. In order to know the specific relationship with arsenite resistance, protein aggregation under other stresses should be shown for the most representative mutants in each class.
3. The authors claim that intracellular arsenic is a direct cause of protein aggregation. This statement is essentially based on their finding that mutants directly affected in arsenic resistance were found in both the enhanced and reduced aggregation groups. However, these mutants also display the expected alterations in intracellular levels of arsenite, which should exacerbate or diminish both direct (i.e. affecting protein folding) and indirect (e.g. through mitochondrial dysfunction) effects of intracellular arsenite in protein aggregation. The authors should tone down their conclusion.
4. Similar screens have been performed using SG reporters (Ohn et al 2008 Buchan et al 2012, Yang et al 2014). Since they support similar conclusions to those presented in the present work, they should be carefully compared and the likely relationships discussed.
Minor points:
1. Does the mutant list include SG core proteins or their transcriptional regulators?
2. Many mutants displaying reduced protein aggregation are involved in key growth processes. Does growth rate per se affect protein aggregation?

Cherkasov V, Hofmann S, Druffel-Augustin S, Mogk A, Tyedmers J, Stoecklin G Bukau B. Coordination of translational control and protein homeostasis during severe heat stress. Curr Biol. 2013 Dec 16; 23(24):2452-62.
Yang X, Shen Y, Garre E, Hao X, Krumlinde D, Cvijović M, Arens C, Nyström T, Liu B, Sunnerhagen P. Stress granule-defective mutants deregulate stress responsive transcripts. PLoS Genet. 2014 Nov 6;10(11):e1004763.
Ohn T, Kedersha N, Hickman T, Tisdale S, Anderson P. A functional RNAi screen links O-GlcNAc modification of ribosomal proteins to stress granule and processing body assembly. Nat Cell Biol. 2008 Oct;10(10):1224-31.
Buchan JR, Kolaitis RM, Taylor JP, Parker R. Eukaryotic stress granules are cleared by autophagy and Cdc48/VCP function. Cell. 2013 Jun 20;153(7):1461-74.

Reviewer 2

Advance summary and potential significance to field

This manuscript describes a genome-wide microcopy screen of arsenite-exposed yeast cells. The screen identified 202 mutants that exhibit increased protein aggregation and 198 mutants with reduced aggregation (in total near 8% of the non-essential genes knockouts tested). Follow-up

© 2021. Published by The Company of Biologists under the terms of the Creative Commons Attribution License (https://creativecommons.org/licenses/by/4.0/).

experiments using selected mutants support lend the notion that translation output (or indirectly transcription output) is an important factor that impacts on the response to arsenite exposure.

The authors and others have previously shown that arsenite treatment induces protein misfolding of newly synthesized proteins and they interpret the identified mutations within this conceptual framework. The data is convincing, yet the manuscript would become more exciting if other insights from the screen were developed somewhat during revision.

Comments for the author

Specific concerns:
1. The large fraction of library knock-out strains that scored in the screen (8% of the non-essential genes tested) questions how specific the screen is to arsenite/metal biology. The authors have great experience in using the Hsp104-GFP library under various conditions, raising the question if the numerous hits isolated can be narrowed down to arsenite-specific biology by comparing it to other datasets? One dataset not based on the HSP04-GFP reporter that may be useful to categorize knockout strains that affect proteostasis is Brandman et al 2012. Cell. Nov 21;151(5):1042-54. Such further analysis of the dataset may strengthen the manuscript.
2. The comparison of the new data on arsenite-induced aggregation with previous resistance and sensitivity screening (Fig. 2D-E) shows significant overlaps linking aggregation to arsenite sensitivity. Nevertheless, the majority of the genes isolated in the respective screens are not overlapping. This is not explained in the manuscript. Are there problems with the screens so that they yield false positives or are other mechanisms at play?
3. Cellular levels of arsenite impacts on the propensity of the heavy metal to induce protein aggregation (Fig. 3). The data is convincing with the exception of ptp2/2D and ycf1D that appear to exhibit at best very modest changes of arsenite levels. Can the authors provide a reference or titration experiment that shows that such small changes of the intracellular arsenite levels impact on protein folding protein aggregation? Moreover, the authors note in the Discussion that priority should be given to systematically measure intracellular arsenite levels in the identified mutants. Although considered for future work, such data would strengthen the study significantly.
4. The screen implicates MSN2 and MSN4 in arsenite biology and translational regulation (Fig 6). The authors find that msn2D msn4D cells are more efficient in reducing translational initiation during arsenite exposure. Msn2 and Msn4 are transcription factors and it is unclear if the effect on translational initiation is related to altered physiology of the msn2D msn4D prior to arsenite addition or if these transcription factors act rapidly post arsenite addition. The time of arsenite exposure in Fig. 6E is not clear in the manuscript and this should be included so that the data can be interpreted. The authors also mention that the upstream signaling network (Snf1 and glucose/cAMP signaling pathways) of Msn2/4 have been identified in the screen (p. 7). How does this connect? Msn2 and Msn4 have a broad repertoire of target genes including the aggregation reporter HSP104 that forms the basis for the screen and the follow-up experiments. Effects of the msn2D msn4D mutations on the reporter should be ruled out. Furthermore, can the authors provide an explanation for how Msn2 and Msn4 are linked to translational control? Translation initiation is typically controlled by amino acid availability and it is probably worth looking at the link to Msn2 and Msn4 (Perhaps PMID: 32411186 is helpful?)

First revision

<u>Author response to reviewers' comments</u>

Reviewer 1 Advance summary and potential significance to field
The paper by Andersson et al describes a screen for genes involved, either directly or indirectly, in protein aggregation by arsenite in yeast. The methods used are appropriate and controls were properly introduced. Although other screens with similar purposes have been already published (see

© 2021. Published by The Company of Biologists under the terms of the Creative Commons Attribution License (https://creativecommons.org/licenses/by/4.0/).

below), the present work focuses on a specific stressor that had not been characterized at a genome-wide level and constitutes a valuable study. In my view, however, there are some major issues that should be addressed before publication is considered.

Reviewer 1 Comments for the author
Major points:
1. Hsp104 co-localizes with Pab1 in SGs after heat shock (Cherkasov et al 2013). Since arsenite also induces SG formation, the co-localization extent of Hsp104 with SG reporter proteins under the specific conditions used by the authors should be shown.

Reply: Co-localization of Hsp104-GFP and Pab1-CFP (SG marker) and Dcp2-RFP (P-body marker) during arsenite stress was investigated by us in a previous report (Jacobson et al (2012) J Cell Sci 125, 5073-5083). The majority of Hsp104-GFP-containing foci did not co-localize with SGs and PBs during As(III) exposure in S. cerevisiae, suggesting that formation of Hsp104-GFP foci and SGs/PBs might be largely distinct. We have included this information in the revised manuscript.
We have also compared our data-sets to sets of mutants with altered SG and PB levels. We conclude that some of the genes involved in PQC during As(III) stress may also be involved in modulating SG assembly/disassembly. This new information is included in the revised manuscript.

2. In order to know the specific relationship with arsenite resistance, protein aggregation under other stresses should be shown for the most representative mutants in each class.

Reply: We have compared our data-sets with other genome-wide data-sets for modulators of SG/PB assembly (Yang et al 2014, Buchan et al 2012), for defects in assembly of large inclusions during heat stress (Babazadeh et al 2019), and mutants that display altered activity of a reporter containing binding sites for the yeast heat shock factor 1 (Brandman et al 2012). Collectively, these analyses suggest that our data-sets contain factors that may act specifically during As(III)-induced protein aggregation as well as general factors acting under proteotoxic stress. This new information is included in the revised manuscript.

3. The authors claim that intracellular arsenic is a direct cause of protein aggregation. This statement is essentially based on their finding that mutants directly affected in arsenic resistance were found in both the enhanced and reduced aggregation groups. However, these mutants also display the expected alterations in intracellular levels of arsenite, which should exacerbate or diminish both direct (i.e. affecting protein folding) and indirect (e.g. through mitochondrial dysfunction) effects of intracellular arsenite in protein aggregation. The authors should tone down their conclusion.

Reply: Point well taken. We toned down the conclusions in the revised manuscript by deleting the section ‘Correlation between intracellular arsenic and protein aggregation’ and incorporating the observations related to arsenic transport and intracellular accumulation to other parts of the text. The corresponding section in the Discussion has been shortened.

4. Similar screens have been performed using SG reporters (Ohn et al 2008, Buchan et al 2012, Yang et al 2014). Since they support similar conclusions to those presented in the present work, they should be carefully compared and the likely relationships discussed.

Reply: We have compared our data-sets with other genome-wide data-sets for modulators of SG/PB assembly (Yang et al 2014, Buchan et al 2012), for defects in assembly of large inclusions during heat stress (Babazadeh et al 2019), and mutants that display altered activity of a reporter containing binding sites for the yeast heat shock factor 1 (Brandman et al 2012). Collectively, these analyses suggest that our data-sets contain factors that may act specifically during As(III)-induced protein aggregation as well as general factors acting under proteotoxic stress. This new information is included in the revised manuscript.

Minor points:
1. Does the mutant list include SG core proteins or their transcriptional regulators?

© 2021. Published by The Company of Biologists under the terms of the Creative Commons Attribution License (https://creativecommons.org/licenses/by/4.0/).

Reply: Some SG and PB components were present in our data-sets (e.g. Pbp1, Dhh1, Xrn1) but most core SG/PB proteins were absent (e.g. Pub1, Pab1, Cdc33, Tif4631 and Tif4632, Edc3, Dcp2). This new information is included in the revised manuscript.

2. Many mutants displaying reduced protein aggregation are involved in key growth processes. Does growth rate per se affect protein aggregation?

Reply: Point well taken. Slow growth is accompanied by a reduction of translation-related proteins (Metzl-Raz et al., 2017) and probably by lower protein synthesis rates. We found a significant overlap between a set of mutants that grow slowly in minimal SC medium and the reduced aggregation set. This suggests that ongoing translation results in As(III)-induced protein aggregation. This new information is included in the revised manuscript.

Reviewer 2 Advance summary and potential significance to field
This manuscript describes a genome-wide microcopy screen of arsenite-exposed yeast cells. The screen identified 202 mutants that exhibit increased protein aggregation and 198 mutants with reduced aggregation (in total near 8% of the non-essential genes knockouts tested). Follow-up experiments using selected mutants support lend the notion that translation output (or indirectly transcription output) is an important factor that impacts on the response to arsenite exposure. The authors and others have previously shown that arsenite treatment induces protein misfolding of newly synthesized proteins and they interpret the identified mutations within this conceptual framework. The data is convincing, yet the manuscript would become more exciting if other insights from the screen were developed somewhat during revision.

Reviewer 2 Comments for the author
Specific concerns:
1. The large fraction of library knock-out strains that scored in the screen (8% of the non-essential genes tested) questions how specific the screen is to arsenite/metal biology. The authors have great experience in using the Hsp104-GFP library under various conditions, raising the question if the numerous hits isolated can be narrowed down to arsenite-specific biology by comparing it to other datasets? One dataset not based on the HSP04-GFP reporter that may be useful to categorize knockout strains that affect proteostasis is Brandman et al 2012. Cell. Nov 21;151(5):1042-54. Such further analysis of the dataset may strengthen the manuscript.

Reply: We have compared our data-sets with other genome-wide data-sets for modulators of SG/PB assembly (Yang et al 2014, Buchan et al 2012), for defects in assembly of large inclusions during heat stress (Babazadeh et al 2019), and mutants that display altered activity of a reporter containing binding sites for the yeast heat shock factor 1 (Brandman et al 2012). Collectively, these analyses suggest that our data-sets contain factors that may act specifically during As(III)-induced protein aggregation as well as general factors acting under proteotoxic stress. This new information is included in the revised manuscript.

2. The comparison of the new data on arsenite-induced aggregation with previous resistance and sensitivity screening (Fig. 2D-E) shows significant overlaps linking aggregation to arsenite sensitivity. Nevertheless, the majority of the genes isolated in the respective screens are not overlapping. This is not explained in the manuscript. Are there problems with the screens so that they yield false positives or are other mechanisms at play?

Reply: Point well taken. We have added text explaining that protein misfolding and aggregation is not the only toxicity mechanism but acts in parallel with previously described toxicity mechanisms such as oxidative stress-induced damage to DNA, lipids and proteins, inhibition of DNA repair and disruption of enzyme function.

3. Cellular levels of arsenite impacts on the propensity of the heavy metal to induce protein aggregation (Fig. 3). The data is convincing with the exception of ptp2/2D and ycf1D that appear to exhibit at best very modest changes of arsenite levels. Can the authors provide a reference or titration experiment that shows that such small changes of the intracellular arsenite levels impact on protein folding protein aggregation? Moreover, the authors note in the Discussion that priority should be given to systematically measure intracellular arsenite levels in the identified mutants. Although considered for future work, such data would strengthen the study significantly.

© 2021. Published by The Company of Biologists under the terms of the Creative Commons Attribution License (https://creativecommons.org/licenses/by/4.0/).

Reply: As suggested by reviewer 1, we toned down the conclusions in the revised manuscript by deleting the section ‘Correlation between intracellular arsenic and protein aggregation’ and incorporating the observations related to arsenic transport and intracellular accumulation to other parts of the text. The corresponding section in the Discussion has been shortened.

4. The screen implicates MSN2 and MSN4 in arsenite biology and translational regulation (Fig 6). The authors find that msn2D msn4D cells are more efficient in reducing translational initiation during arsenite exposure. Msn2 and Msn4 are transcription factors and it is unclear if the effect on translational initiation is related to altered physiology of the msn2D msn4D prior to arsenite addition or if these transcription factors act rapidly post arsenite addition. The time of arsenite exposure in Fig. 6E is not clear in the manuscript and this should be included so that the data can be interpreted. The authors also mention that the upstream signaling network (Snf1 and glucose/cAMP signaling pathways) of Msn2/4 have been identified in the screen (p. 7). How does this connect? Msn2 and Msn4 have a broad repertoire of target genes including the aggregation reporter HSP104 that forms the basis for the screen and the follow-up experiments. Effects of the msn2D msn4D mutations on the reporter should be ruled out. Furthermore, can the authors provide an explanation for how Msn2 and Msn4 are linked to translational control? Translation initiation is typically controlled by amino acid availability and it is probably worth looking at the link to Msn2 and Msn4 (Perhaps PMID: 32411186 is helpful?)

Reply: The exposure time in Fig. 6E (now 5E) was 1 hour and this is now clearly indicated in the figure legend.

We demonstrate that the absence of Msn2 and Msn4 promotes strong translation inhibition in response to As(III). Notably, translation activity is similar in wild type and msn2/msn4 cells prior to stress and MSN2/MSN4 deletion does not affect Hsp104 and Hsp70 protein levels or intracellular arsenic accumulation. Hence, msn2/ msn4 is not pre-adapted to stress by increased folding capacity and/or lower translation activity but responds to As(III) by robust translation inhibition. Efficient translation inhibition is likely responsible for the diminished aggregation levels observed in msn2/msn4, as well as for its HygB and As(III) resistance. The targets of Msn2/Msn4 that regulate translation inhibition remain to be identified. This is clearly stated in the revised manuscript.

The broad network of cellular systems identified here provides a valuable resource and a framework for dedicated follow-up studies of the molecular underpinnings of arsenic toxicity and pathogenesis. Snf1 and PKA could impact on proteostasis under As(III) exposure in several ways. Beside Msn2/Msn4, Snf1 and PKA regulate various targets via phosphorylation resulting in altered expression of a wide variety of genes. For example, Snf1 regulates the heat shock factor Hsf1 and aids in the recruitment of the SAGA complex and RNA Polymerase II to specific target promoters, whilst PKA affects expression of protein biosynthesis genes. Whether Snf1/PKA affect proteostasis under As(III) exposure via Msn2/Msn4 or other targets is certainly worth pursuing. Likewise, exploring the correlation between amino acid availability, translational control and the role of Msn2/Msn4 is interesting and certainly worth pursuing. However, we feel that these suggestions are better suited in dedicated follow-up studies.

Second decision letter

MS ID#: JOCES/2020/258338

MS TITLE: Genome-wide imaging screen uncovers molecular determinants of arsenite-induced protein aggregation and toxicity

AUTHORS: Stefanie Andersson, Antonia Romero, Joana Isabel Rodrigues, Sansan Hua, Xinxin Hao, Therese Jacobson, Vivien Karl, Nathalie Becker, Arghavan Ashouri, Sebastien Rauch, Thomas Nystrom, Beidong Liu, and Markus J Tamas
ARTICLE TYPE: Research Article

© 2021. Published by The Company of Biologists under the terms of the Creative Commons Attribution License (https://creativecommons.org/licenses/by/4.0/).

I am happy to tell you that your manuscript has been accepted for publication in Journal of Cell Science, pending standard ethics checks.

Reviewer 1

Advance summary and potential significance to field

The authors have added new information that will help the reader to incorporate this interesting work in the existing literature on the mechanisms of protein aggregation.

Comments for the author

The authors have fully addressed all my questions and suggestions, and have added new information that will help the reader to incorporate this interesting work in the existing literature on the mechanisms of protein aggregation.

Reviewer 2

Advance summary and potential significance to field

Following revision with the the addition of further analysis of the screening data, the manuscript now provides a rich source of information regarding heavy metal toxicity and its impact on proteostasis.

Comments for the author

All my concerns have been addressed.

© 2021. Published by The Company of Biologists under the terms of the Creative Commons Attribution License (https://creativecommons.org/licenses/by/4.0/).
